# Supramolecular Radiosensitizer Based on Hypoxia‐Responsive Macrocycle

**DOI:** 10.1002/advs.202104349

**Published:** 2022-01-07

**Authors:** Xiaoxue Hou, Yu‐Xuan Chang, Yu‐Xin Yue, Ze‐Han Wang, Fei Ding, Zhi‐Hao Li, Hua‐Bin Li, Yicheng Xu, Xianglei Kong, Fan Huang, Dong‐Sheng Guo, Jianfeng Liu

**Affiliations:** ^1^ CAMS Key Laboratory of Radiopharmacokinetics for Innovative Drugs Institute of Radiation Medicine Chinese Academy of Medical Sciences & Peking Union Medical College Tianjin 300192 P. R. China; ^2^ College of Chemistry Key Laboratory of Functional Polymer Materials (Ministry of Education) State Key Laboratory of Elemento‐Organic Chemistry National Demonstration Center for Experimental Chemistry Education Nankai University Tianjin 300071 P. R. China

**Keywords:** calixarene, drug delivery, radiotherapy, supramolecular chemistry, tumor hypoxia

## Abstract

Radiotherapy (RT) has been viewed as one of the most effective and extensively applied curatives in clinical cancer therapy. However, the radioresistance of tumor severely discounts the radiotherapy outcomes. Here, an innovative supramolecular radiotherapy strategy, based on the complexation of a hypoxia‐responsive macrocycle with small‐molecule radiosensitizer, is reported. To exemplify this tactic, a carboxylated azocalix[4]arene (CAC4A) is devised as molecular container to quantitatively package tumor sensitizer banoxantrone dihydrochloride (AQ4N) through reversible host–guest interaction. Benefited from the selective reduction of azo functional groups under hypoxic microenvironment, the supramolecular prodrug CAC4A•AQ4N exhibits high tumor accumulation and efficient cellular internalization, thereby significantly amplifying radiation‐mediated tumor destruction without appreciable systemic toxicity. More importantly, this supramolecular radiotherapy strategy achieves an ultrahigh sensitizer enhancement ratio (SER) value of 2.349, which is the supreme among currently reported noncovalent‐based radiosensitization approach. Further development by applying different radiosensitizing drugs can make this supramolecular strategy become a general platform for boosting therapeutic effect in cancer radiotherapies, tremendously promising for clinical translation.

## Graphical abstract

The authors propose a novel concept of supramolecular radiotherapy to enhance the antitumor activity of radiation by host–guest strategy. Through complexing radiosensitive drugs within hypoxia‐responsive macrocyclic hosts to form supramolecular formulation, the supramolecular radiotherapy strategy significantly amplifies radiation‐mediated tumor destruction and reduces undesirable damage to normal tissues.

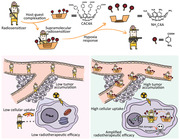

## 1 Introduction

Radiotherapy (RT), as a mainstream modality in the field of noninvasive cancer treatment, kills cancerous cells via direct or indirect DNA damages upon high‐energy ionization radiation rays.^[^ ^1^ ^]^ According to the statistics, over half of cancer patients benefit from RT alone or combined with other treatment modalities including surgery, chemotherapy or immunotherapy.^[^ ^2^ ^]^ However, the inherent and acquired radioresistance of tumors has been a longstanding obstacle for achieving ideal therapeutic outcomes in clinic.^[^ ^3^ ^]^ Thus, considerable efforts have been dedicated to develop radiosensitizers, such as small‐molecule drugs,^[^ ^4^ ^]^ proteins,^[^ ^5^ ^]^ peptides,^[^ ^6^ ^]^ miRNAs,^[^ ^7^ ^]^ siRNAs,^[^ ^8^ ^]^ and various nanomaterials,^[^ ^9^ ^]^ which can improve radiotherapeutic efficiency by concentrating radiation energy in tumors. One of the most significant standards for assessing a radiosensitizer is sensitizer enhancement ratio (SER) value, which is a universally accepted indicator for comparison with the radiosensitizationcapability of various radiosensitizers.^[^ ^10^ ^]^Nowadays, radiosensitive small‐molecule drugs have been one of the most commendable candidates due to their advantages including definite structures, synthetic flexibility, mature technology and lower cost.^[^ ^11^ ^]^ Nevertheless, randomized accumulation and rapid metabolism results in small‐molecule drugs with low SER value, which was severely impeding their widespread clinical application. Although several drug‐delivery systems such as liposomes,^[^ ^12^ ^]^ albumin,^[^ ^13^ ^]^ inorganic,^[^ ^14^ ^]^ and polymeric nanoparticles^[^ ^15^ ^]^ have been implemented to shuttle radiosensitive small‐molecule drugs to the tumor sites, there still remains a huge challenge to develop facile and robust strategy for achieving higher SER value to boost cancer radiotherapy without complicated purification and unrepeatable production.

Supramolecular chemistry recently has attracted tremendous attention in advancing the tumor‐targeted therapy.^[^ ^16^ ^]^ Various types of macrocyclic hosts including cyclodextrins,^[^ ^17^ ^]^ calixarenes,^[^ ^18^ ^]^ cucurbiturils,^[^ ^19^ ^]^ and pillararenes^[^ ^20^ ^]^ have been utilized as molecular vessel to ferry therapeutic drugs into tumors, thereby enhancing the therapeutic efficacy and/or alleviating side effects. Unlike traditional nanoscale drug‐delivery systems, supramolecular macrocycles not only show operational simplicity but also possess well‐defined molecular structures and molecular weight, which can guarantee the batch‐to‐batch consistency through rigorous manufacturing process and careful quality control. Moreover, the unique properties of tunable cavity size and easy modification empower these macrocyclic hosts with fascinating molecular recognition ability, thereby quantitatively binding a wide range of drug guests.^[^ ^21^ ^]^ As a result, macrocycles have been widely engaged in supramolecular chemotherapy, chemodynamic therapy, activatable photodynamic therapy, gene therapy, immunotherapy and bioimaging.^[^ ^22^ ^]^ In view of these significant achievements, there is a reasonable and promising prospect that supramolecular macrocycles can be available for radiotherapy with improved SER value and treatment outcome. An ideal supramolecular strategy for effective cancer radiotherapy should meet the following features: i) high binding affinity between macrocyclic host and radiosensitive drugs to avoid undesired off‐target leakage; ii) efficient delivery to tumor for radiosensitive drugs by targeting distinctive characteristic of tumor microenvironment; and iii) enhanced cellular internalization and intracellular drug release to exert optimal radiosensitizing effect. As a result, the key challenge emerges to be designing smart supramolecular macrocycles with robust binding capability, stimuli‐responsibility, as well as cellular uptake.

**Scheme 1:**
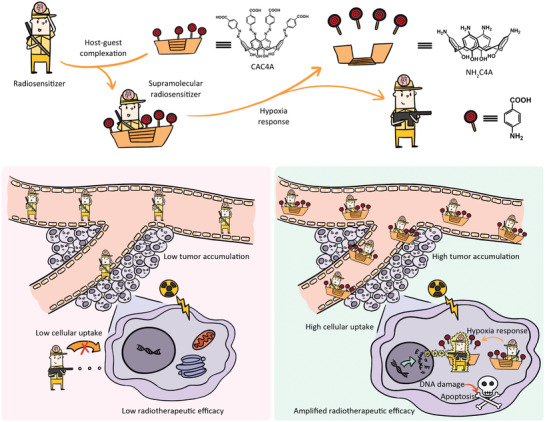
Schematic illustration of the supramolecular radiotherapy strategy based on host–guest chemistry and the corresponding enhanced radiotherapeutic efficiency mechanism.

Herein, we propose a supramolecular radiotherapy based on hypoxia‐responsive macrocycle to enhance the antitumor activity of radiation (**Scheme** **1**). Carboxylated azocalix[*n*]arenes (CAC*n*As, *n* = 4, 5, 6, 8) and banoxantrone dihydrochloride (AQ4N) were selected as the model supramolecular macrocycle and radiosensitive small‐molecule drug, respectively. After screening from CAC*n*A family, carboxylated azocalix[4]arene (CAC4A) with four phenolic units were found to afford the highest binding affinity toward AQ4N, demonstrating its most appropriate ability as a molecular container for AQ4N and formation of most stable host–guest complex (CAC4A•AQ4N). Moreover, the azo groups of CAC4A can be readily reduced to amino groups under hypoxic microenvironment of tumor tissue, and this will lead to the decrease of binding affinity between AQ4N and calixarene and the dissociation of CAC4A•AQ4N, thereby realizing tumor targeted release of AQ4N. As a result, the complexation of CAC4A significantly advanced tumor accumulation and cellular internalization of radiosensitive AQ4N, thus greatly amplifying radiotherapeutic efficacy and minimizing undesirable damage to normal tissues. Surprisingly, the CAC4A•AQ4N complex achieves an ultralarge SER value of 2.349, which is the highest among currently reported noncovalent‐based radiosensitization approach.^[^ ^23^ ^]^ Therefore, this work realizes the enhanced cancer radiotherapy based on supramolecular macrocycle for the first time, providing new insights for the development of advanced radiotherapies for cancer treatments in a simple way.

## 2 Result and Discussion

**Figure 1:**
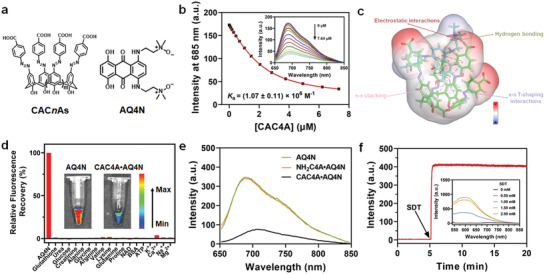
Molecular recognition and in vitro drug release simulation. a) Molecular structures of CAC*n*As (*n* = 4, 5, 6, 8) and AQ4N. b) Direct fluorescence titration of AQ4N (2.00 × 10^−6^ m) with CAC4A in PBS buffer (10 × 10^−3^ m, pH = 7.4) at 25 °C, *λ*_ex_ = 610 nm. Inset: The associated titration curve at *λ*_em_ = 685 nm was fitted according to the 1:1 binding stoichiometry. c) Interactions and corresponding electrostatic potential in the final pose of CAC4A•AQ4N complex after a 100 ns molecular dynamics simulation. C atoms of CAC4A and AQ4N are shown in green and cyan, respectively. Oxygen and nitrogen atoms in this complex are colored by red and blue, respectively. *π*–*π* stacking and *π*–*π* T‐shaping interactions are highlighted in pink and blue dotted lines, respectively. Electrostatic and hydrogen bonding interactions are colored by red and green dotted lines, respectively. The hydrogen‐bonding criteria are i) the angle C—H···O > 135° and ii) the distance C···O < 3.5 Å. d) Fluorescent responses of CAC4A•AQ4N (30.00/10.00 × 10^−6^ m) when adding different biomolecules in blood in PBS (10 × 10^−3^ m, pH = 7.4), 25 °C, *λ*_ex_ = 610 nm. e) Fluorescence spectra of AQ4N (10.00 *µ*M), CAC4A•AQ4N (30.00/10.00 *µ*M) and NH_2_C4A•AQ4N (30.00/10.00 × 10^−6^ m) in PBS buffer (10 × 10^−3^ m, pH = 7.4), 25 °C. f) Fluorescence intensity at 590 nm of CAC4A•AQ4N (30.00/10.00 × 10^−6^ m) as a function of time following the addition of SDT (1.00 × 10^−3^ m) in PBS buffer (10 × 10^−3^ m, pH = 7.4), 37 °C, *λ*_ex_ = 517 nm. Inset: Fluorescence spectra of CAC4A•AQ4N (30.00/10.00 × 10^−6^ m) after addition of various concentrations of SDT (up to 2.50 × 10^−3^ m) in PBS buffer (10 × 10^−3^ m, pH = 7.4), 25 °C, *λ*_ex_ = 517 nm.

As a low toxicity drug, AQ4N has recently drawn great attentions in sensitizing tumor to radiotherapy.^[^ ^24^ ^]^ However, AQ4N exhibits salt‐like properties, and bestows a series of insurmountable obstacles such as excessive aqueous solubility, low cellular uptake and rapid in vivo elimination.^[^ ^25^ ^]^ Accordingly, we first set out to find a proper supramolecular macrocycle as molecular container for delivering AQ4N. A suitable molecular container should be able to 1) bind AQ4N with high binding affinity to avoid undesired off‐target leakage and blood clearance, 2) permeate cell membrane for intracellular delivery, 3) respond to the microenvironment of tumor tissue for targeted cargo release, 4) quench the fluorescence of AQ4N for tumor‐specific imaging, and 5) induce minimum systemic cytotoxicity for in vivo application. These criteria made us reminisce about the CAC4A, based on which we designed a versatile anticancer drug delivery platform consisting of the complex of CAC4A and drug molecules.^[^ ^26^ ^]^ The azo groups on CAC4A can be reduced readily under the action of azoreductase to form aniline derivatives in the hypoxic environment of tumor tissue, leading to targeted delivery of cancer drugs for optimized cancer therapy. We envisioned that a carboxylated azocalixarene like CAC4A could bind with AQ4N with high affinity and serve as a molecular container due to ionic interactions between carboxylates and amine oxides and *π*–*π* stacking between conjugated systems, and that the intrinsic fluorescence of AQ4N can be quenched via photoinduced electron transfer when encapsulated. Based on our hypothesis, four carboxylated azocalixarenes (CAC*n*As, *n* = 4, 5, 6, 8) were designed and synthesized (**Figure** **1**a and Scheme S1, Supporting Information). The binding behaviors of CAC*n*As with AQ4N were determined by direct fluorescence titrations (Figures 1b and Figures S7–S9, Supporting Information). Among all calixarenes tested, CAC4A exhibits the strongest 1:1 binding toward AQ4N (*K*_a_ = (1.07 ± 0.11) × 10^6^ M^−1^), making it the most suitable candidate as the carrier for AQ4N. Having confirmed the strong interaction between CAC4A and AQ4N in the excited state by fluorescence spectrophotometer, we then employed UV–vis spectroscopy to verify the interaction between them in the ground state. As shown in Figure S13 (Supporting Information), CAC4A displayed a broad absorption peak around 400 nm, while that for AQ4N was around 600 nm. The absorption spectrum of CAC4A•AQ4N simultaneously displayed characteristic peaks of CAC4A and AQ4N while an obvious redshift at 600 nm was observed, indicating the formation of CAC4A•AQ4N complex. Therefore, CAC4A was used for further experiments.

We then sought to understand the mechanism behind the strong binding between CAC4A and AQ4N. 100 ns molecular dynamics (MD) simulation was adopted to study the binding geometry (Figure 1c and Figure S15, Supporting Information). As expected, the hydroquinone part of AQ4N located at the center of the cavity of CAC4A all the time, while the amine oxide parts of the former were at the upper rim of the latter. Multiple *π*‐*π* stacking interactions were also found in the CAC4A•AQ4N complex. Furthermore, electrostatic interactions formed between oxygen atom of carboxyl group in CAC4A and nitrogen atom of amine oxide group and hydrogen bonding formed between oxygen atom and hydrogen atom of methylene group in AQ4N helped further stabilize the guest. Collectively, electrostatic, hydrogen bonding and *π*–*π* stacking interactions synergistically led to the stable complexation between CAC4A and AQ4N.

Since supramolecular host–guest pairs may suffer from undesired competition from biomolecules during the blood circulation, it is essential to evaluate the anti‐interference ability of CAC4A•AQ4N in the complicated biological environment. We therefore utilized major biological species in blood to challenge the integrity of CAC4A•AQ4N. As shown in Figure 1d, there was almost no fluorescence recovery from CAC4A•AQ4N when incubated with various biomolecules in blood such as glutathione, urea, glucose, creatinine, alanine, glycine, arginine, valine, lysine, glutamine, proline, nicotinamide adenine dinucleotide (NAD), bovine serum albumin (BSA), adenosine triphosphate (ATP), K^+^, Ca^2+^, Na^+^, and Mg^2+^, demonstrating that the CAC4A•AQ4N can commendably maintain its structural integrity and effectively avoid AQ4N leakage during blood circulation. Moreover, no appreciable fluorescence regeneration could be observed from CAC4A•AQ4N incubated with mouse serum (MS) (Figure 1d, inset), further confirming the great stability of CAC4A•AQ4N under normal physiological environment. However, the heme found in serum may reduce the N‐oxides under hypoxic conditions.We then conducted to detect the integrity of CAC4A, AQ4N and CAC4A•AQ4N in the presence of heme by incubating CAC4A, AQ4N and CAC4A•AQ4N in complete medium containing heme under hypoxic conditions. CAC4A, AQ4N and CAC4A•AQ4N in serum‐free media were employed for better comparison. As shown in Figure S16a (Supporting Information), no obvious absorption peak of reduction product NH_2_C4A and AQ4 was displayed after incubating CAC4A and AQ4N with complete medium containing heme, respectively. And the fluorescence spectra of AQ4N and CAC4A•AQ4N was shown in Figure S16b (Supporting Information). Negligible fluorescence recovery could be observed from CAC4A•AQ4N after treated with complete medium containing heme. Therefore, the reduction of CAC4A, AQ4N and CAC4A•AQ4N complex were induced by the tumor hypoxic environment rather than the heme in serum.

Liposomes like dipalmitoylphosphatidylcholine (DPPC) are traditionally used as delivery agent for AQ4N.^[^ ^27^ ^]^ In order to compare the loading difference between CAC4A and liposomes, AQ4N@DPPC was prepared according to standard protocol.^[^ ^28^ ^]^ Briefly, 0.50 mmol DPPC was dissolved in chloroform and dried on a rotatory evaporator. The formed lipid film was then hydrated with 1.00 mL 0.50 × 10^−3^ m AQ4N at 45 °C. The obtained liposome was then extruded through a 200 nm polycarbonate filter at 45 °C for 20 times, and the unencapsulated AQ4N was removed via a sephadex G‐25 column. The encapsulation percentage (EN%) for AQ4N@DPPC was detected to be 0.2% (Figure S17, Supporting Information). The unsuccessful encapsulation of AQ4N@DPPC might be attributed to the relatively low concentrations of AQ4N and DPPC used. As well known, the physical embedding process depends highly on concentrations. CAC4A•AQ4N was prepared by simply mixing the PBS 7.4 solutions of CAC4A and AQ4N together to reach the final concentrations of 0.50 × 10^−3^ m, respectively, which were the same as that of AQ4N@DPPC. The EN% was calculated to be as high as 95.8% according to the binding affinity. Relative to drug delivery systems relying on physical embedding or absorption, the present supramolecular delivery system provides the exact cavity‐loading pattern via host–guest inclusion and quantified host–guest binding constant, and thereby owns advantages of quantitative loading, high EN%, reproducibility mediated by the corresponding host–guest binding affinities.^[^ ^29^ ^]^ Moreover, the host–guest loading process is very mild, simple and repeatable, without need to introduce any organic solvents and extra laborious steps.

CAC4A was reported to be reduced under the action of DT‐diaphorase, an azoreductase that is overexpressed and activated only in the hypoxic environment of many tumors, and form its aniline derivative aminocalix[4]arene (NH_2_C4A) with fast kinetics.^[^ ^26^, ^30^ ^]^ In order for the molecular container to sufficiently release the encapsulated cargo, it is a key prerequisite for the reduced host NH_2_C4A to have low binding affinity toward AQ4N. Direct fluorescence titration results (Figure S11, Supporting Information) showed that the binding constant of NH_2_C4A to AQ4N was two orders of magnitude lower (*K*_a_ = (1.16 ± 0.23) × 10^4^ M^−1^) than that of CAC4A. The distinct decrease in binding affinity for the guest molecules revealed that the packaged drugs AQ4N could be readily released from CAC4A host after the hypoxic reduction, with its intrinsic fluorescence recovered (Figure 1e). Thus, sodium dithionite (SDT) was used as a mimicking reagent of azoreductase in tumor and added into the CAC4A•AQ4N solution, followed by monitoring the change of fluorescence intensity of the solution with time. As seen in Figure 1f, upon the addition of SDT, the fluorescence intensity remarkably increased within a short time period. Moreover, the degree of fluorescence enhancement was proportional to the concentration of SDT (Figure 1f, inset). Furthermore, AQ4N is known as an attractive prodrug that is activated to the toxic metabolite AQ4 in hypoxic tumor regions, which is confused that hypoxia‐responsive drug release may base on AQ4N‐to‐AQ4 conversion under hypoxic condition. We then evaluated the encapsulation of CAC4A toward AQ4. Direct fluorescence titration results (Figure S10, Supporting Information) showed that CAC4A exhibited also a high binding affinity with AQ4 ((3.26 ± 0.82) × 10^6^ M^−1^), avoiding undesired leakage in physiological environment, and this indicated that the hypoxia‐responsive drug release is not based on AQ4N‐to‐AQ4 conversion under hypoxic condition. In order for the molecular container to sufficiently release the encapsulated cargo, it is a key prerequisite for the reduced host NH_2_C4A to have a low binding affinity with AQ4. Direct fluorescence titration results (Figure S12, Supporting Information) showed that the binding constant of NH_2_C4A with AQ4 was two orders of magnitude lower (NH_2_C4A•AQ4: *K*_a_ = (1.48 ± 0.10) × 10^4^ M^−1^) than that of CAC4A. The distinct decrease in binding affinities revealed that the packaged drugs could be readily released from CAC4A host after the hypoxic reduction. The results revealed that the hypoxia selectivity was primarily derived from the hypoxia response of CAC4A. Therefore, CAC4A•AQ4N could favorably achieve drug unloading under the hypoxic environment.

The hypoxia response of CAC4A•AQ4N was further validated by incubating cancer cells with the as prepared supramolecular prodrug under normoxia and hypoxia conditions. As shown in Figure S18 (Supporting Information), incubation of cancer cells with CAC4A•AQ4N under hypoxia exhibited remarkable stronger fluorescence signals than those under normoxia. The increased fluorescence manifested the outstanding hypoxia responsive drug release performance of CAC4A•AQ4N, which could be attributed to the removal of hypoxia sensitive azo groups in CAC4A. Furthermore, the time‐dependent cellular internalization of free AQ4N and CAC4A•AQ4N was then investigated under the hypoxia condition. From Figure S19 (Supporting Information), the fluorescence signal in cells treated with free AQ4N was still very weak even if the incubation time was extended to 24 h. For the CAC4A•AQ4N treated cells, on the contrary, the fluorescence intensity dramatically increased with time and the localization of fluorescence signal changed from cytosol to nucleus during the 24 h incubation. This indicated that the AQ4N released by hypoxia‐responsive CAC4A gradually converted to its activated form AQ4, which could intercalate into DNA. Moreover, the remarkable stronger fluorescence signals of hypoxic cells incubated with CAC4A•AQ4N than that of AQ4N suggested that CAC4A was able to facilitate the delivery of drugs and increase their accumulation in cancer cells. We then conducted the endocytosis inhibitor assays to further investigate the endocytosis mechanisms of CAC4A•AQ4N. As shown in Figure S20 (Supporting Information), the cellular uptake efficiency of AQ4N and CAC4A•AQ4N were both reduced by low temperature (4 °C) treatment, which was attribute to the decrease of membrane fluidity. Moreover, negligible reduction of fluorescence intensity was observed in sodium azide‐pretreated cells after incubation of AQ4N and CAC4A•AQ4N, which suggested that AQ4N and CAC4A•AQ4N were internalized by 4T1 cells by an energy‐independent passive diffusion process.

**Figure 2:**
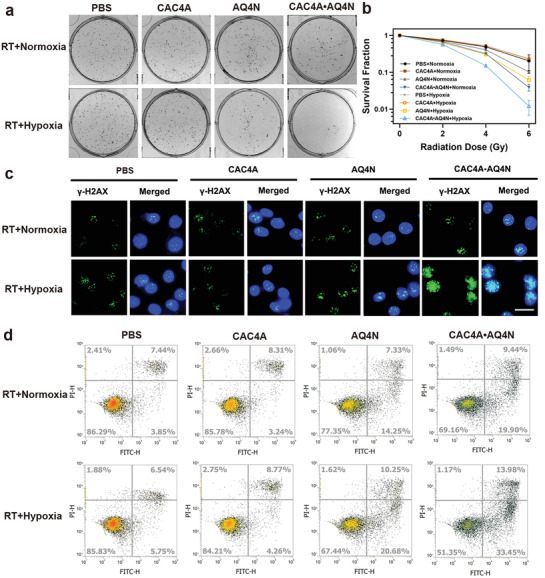
Radiosensitization effect of CAC4A•AQ4N in vitro. a) Colony formation of 4T1 cells treated with CAC4A, AQ4N, CAC4A•AQ4N at radiation dosage of 6 Gy. b) Clonogenic survival curves of 4T1 cells treated with CAC4A, AQ4N, CAC4A•AQ4N at different radiation dosage of 0, 2, 4, 6 Gy. c) Immunofluorescent *γ*‐H2AX staining of 4T1 cells treated with different interventions at radiation dosage of 6 Gy. Scale bar, 50 µm. d) Apoptosis ratios of 4T1 cells treated with different formulations at radiation dosage of 6 Gy were analyzed by flow cytometry. Data are presented as mean ± s.d. (*n* = 3).

Having confirmed the enhanced cellular uptake ability of CAC4A•AQ4N complex, we next evaluated the cytotoxicity of CAC4A•AQ4N for cancer cells by MTT assay. As shown in Figure S21 (Supporting Information), AQ4N and CAC4A•AQ4N exhibited negligible cytotoxicity to 4T1 cells at concentrations ranging from 0 to 200 × 10^−6^ m, which is critical for ideal radiosensitizer to be nontoxic without *γ*‐ray radiation. We next surveyed their radiosensitizing efficacy for cancer cells by clonogenic formation assay, which is considered as a “gold standard” to evaluate the radiosensitization effect of drugs. As shown in **Figure** **2**a,b and Figure S22 (Supporting Information), after ionizing radiation treatment, there were similar trends of colony formation of 4T1 cells for control and CAC4A groups even under the hypoxia conditions, representing the negligible radiation sensitivity of CAC4A. On the contrary, AQ4N+RT treatment exhibited reduced colony formation rates compared to the control groups, corresponding to the radiation‐induced antiproliferation effects of free AQ4N. More importantly, the suppressive ability on colony formation of CAC4A•AQ4N+RT groups were obviously better than that of AQ4N+RT treatments, especially in hypoxic environment, indicating the eminent radiosensitization effect of CAC4A•AQ4N in vitro. It is worth noting that CAC4A•AQ4N complex could achieve the same treatment effect at markedly lower radiation dose, which contributed to relieve the side effects of radiation. Furthermore, based on the clonogenic survival curves (Figure 2b), it was calculated that the SER value of CAC4A•AQ4N strategy was as high as 2.349 (Table S1, Supporting Information), which was the maximum among all the reported noncovalent‐based radiosensitization methods.

It is well known that ionizing radiation damage is closely related to DNA lesions. Immunofluorescent labeling of *γ*‐H2AX, a signaling molecule of DNA damage, was then conducted to evaluate the extent of DNA double‐strand breaks (Figure 2c and Figure S23, Supporting Information). Consistent with the clonogenic survival assay results, strongest *γ*‐H2AX foci with green fluorescence in cell nucleus were detected toward hypoxia 4T1 cells incubated with CAC4A•AQ4N upon ionizing radiation exposure, further proving the potent radiotherapy enhancement effect of CAC4A•AQ4N complex. Meanwhile, Annexin V‐fluoresceine isothiocyanate and propidium iodide double staining assay was carried out by flow cytometry to assess the effect of different treatments on cell apoptosis (Figure 2d and Figure S24, Supporting Information). When exposed to the radiation or incubated in hypoxic environment, the total apoptosis ratio of cells treated with CAC4A•AQ4N complex dramatically increased to 29.3% and 35.8%, respectively, which was much higher than that of AQ4N treatment. Moreover, hypoxic cells treated with CAC4A•AQ4N under ionizing radiation exhibited the most significant apoptosis than other groups. All these results clearly evidenced that CAC4A•AQ4N could efficiently amplify the radio‐therapeutic effect under hypoxia condition.

**Figure 3:**
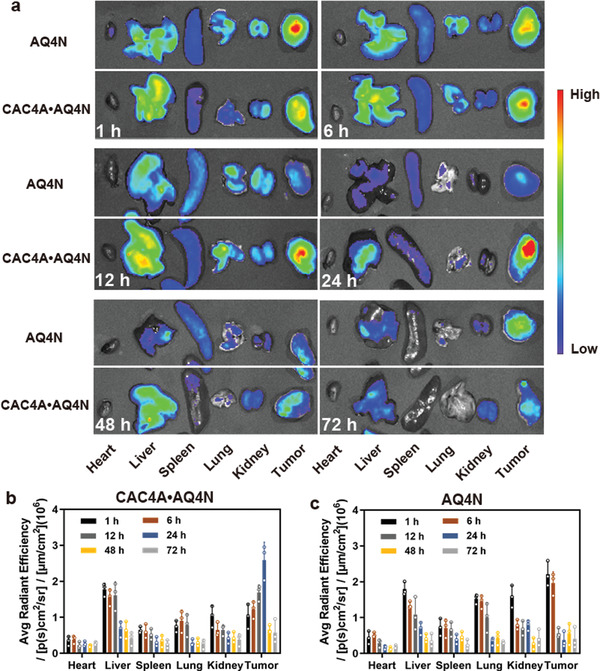
Enhanced tumor accumulation of CAC4A•AQ4N. a) Ex vivo fluorescence images of major organs (heart, liver, spleen, lung, and kidney) and tumor collected from mice at different times (1, 6, 12, 24, 48, 72 h) postinjection of AQ4N and CAC4A•AQ4N. Quantitative analysis of fluorescence signals in major organs and tumors of mice treated with b) CAC4A•AQ4N and c) AQ4N. Data are presented as mean ± s.d. (*n* = 3).

For an enhanced radiotherapy, high accumulation of radiosensitive drugs into tumor tissues was essential to exert optimal radiosensitizing effect. For this study, CAC4A•AQ4N and free AQ4N were intravenously injected into 4T1‐bearing mice and the intrinsic fluorescence of AQ4N was tracked by an *ex vivo* imaging system. As shown in **Figure** **3**a, the fluorescence signals were remarkably boosted in the tumor of mice treated with CAC4A•AQ4N and reached maximum at 24 h postinjection, indicating the successful release and significant accumulation of AQ4N under the tumor hypoxic microenvironment. In contrast, mice administrated with free AQ4N showed weak fluorescence signals in the tumors over time, which was corresponding to the rapid metabolism of small molecule drugs AQ4N from liver and kidney. Similar results were also investigated from the quantitative analysis (Figures 3b,c) and in vivo imaging (Figure S25, Supporting Information), indicating that CAC4A could alter the biodistribution of AQ4N and provide commendable tumor accumulation capacity for the drug.

**Figure 4:**
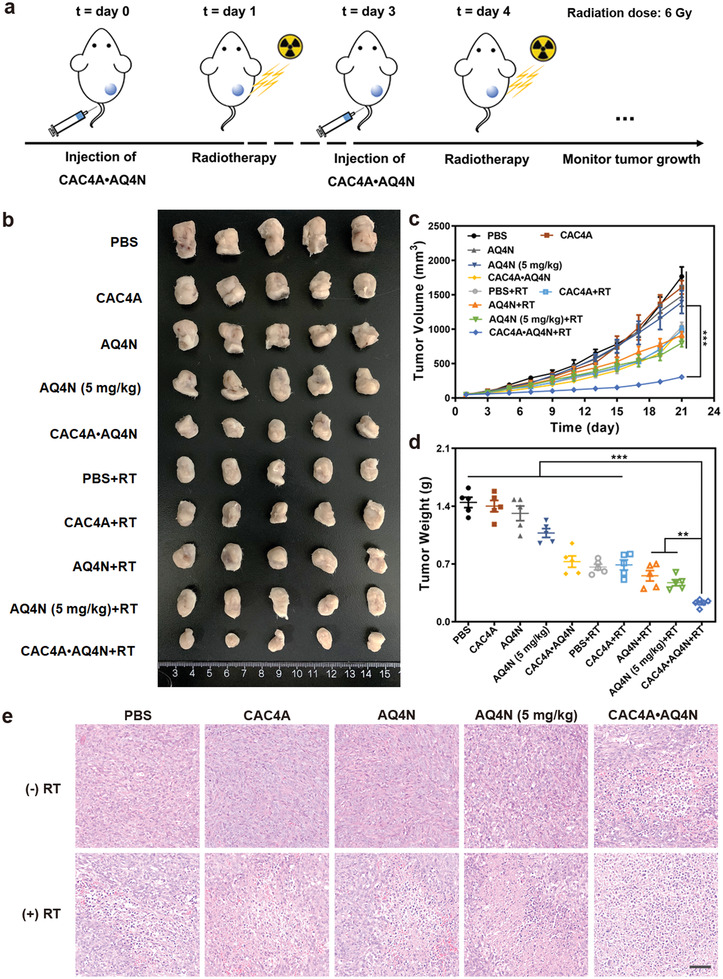
Antitumor efficiency of CAC4A•AQ4N in vivo. a) Schematic illustration experiment design for radiotherapy. Radiation dose: 6 Gy. b) The image of tumors dissected from mice with different treatments. c) Tumor volume growth curves of mice treated with various interventions. d) Tumor weight changes of the mice after different treatments. e) H&E staining analysis of tumor sections from mice after various treatments. Scale bar, 50 µm. Data are presented as mean ± s.d. (*n* = 5). *p*‐values were calculated by ANOVA analysis: **p < 0.01, ***p < 0.001.

Encouraged by the above results, we next investigated the radiotherapeutic efficiency of CAC4A•AQ4N in vivo using mouse tumor model. The BALB/c mice bearing with 4T1 tumor were randomly divided into 10 groups and intravenously injected with different formulations twice on day 0 and day 3, followed by receiving *γ*‐ray radiation at 24 h postinjection or not and simultaneous monitoring the tumor volumes at a 1 d interval over 21 d (**Figure** **4**a). As presented in Figure 4c, the PBS group and CAC4A group showed a rapid increase of tumor volumes, indicating that the macrocycle CAC4A was unable to frustrate the tumor growth. Compared to the PBS group, there was only slight suppression for tumor growth when treated with AQ4N alone even at a high dose of 5 mg kg^−1^, while CAC4A•AQ4N displayed a moderate improved tumor growth inhibition, which was ascribed to the enhanced tumor accumulation and cellular internalization of AQ4N assisted by CAC4A. The mice treated RT alone also exhibited limited inhibition for tumor proliferation and CAC4A could not potentiate the treatment effect under the radiation. Meanwhile, AQ4N+RT group showed negligible tumor suppression enhancement compared with RT alone even at high concentration of AQ4N (5 mg kg^−1^), owing to the fact that poor biodistribution of AQ4N resulted in the discounted treatment outcome. In contrast, combination of CAC4A•AQ4N with RT significantly depressed the tumor growth and the average tumor volume was much smaller than that of other groups, which firmly demonstrates that CAC4A•AQ4N could pronouncedly boost the antitumor effect of radiotherapy in vivo. These results were further validated with the images of tumor dissected from mice (Figure 4b) and the comparison of the tumor weights (Figure 4d). Hematoxylin and eosin (H&E) staining and terminal deoxynucleotidyl transferase dUTP nick end labeling (TUNEL) assay were further performed to assess the therapeutic efficacy of CAC4A•AQ4N. From H&E analysis, there were extensive cells with apoptotic features including condensed nuclei and cell shape changes in tumor sections from CAC4A•AQ4N+RT treated mice, whereas unconspicuous apoptosis could be observed from other groups (Figure 4e). As shown in Figure S26a (Supporting Information), stronger TUNEL signal intensities were observed in CAC4A•AQ4N+RT group, suggesting the improved radiotherapy outcome under the action of CAC4A•AQ4N. To further assess the in vivo antiproliferation effect after different interventions, immunofluorescence Ki67 staining was conducted. As shown in Figure S26b (Supporting Information), the most significant reduced fluorescence intensities were observed in CAC4A•AQ4N+RT group compared with other treatments, indicating that CAC4A•AQ4N could effectively enhance therapeutic efficacy by restraining tumor growth. Therefore, our results clearly indicated that the host–guest complexation between supramolecular macrocycle and radiosensitive drugs indeed could trigger superior radiotherapy compared with drugs alone.

Biosafety, as a pivotal concern for biomedical application, was then carried out to evaluate the clinical potential of our strategy. From Figure S27a (Supporting Information), CAC4A, AQ4N, and CAC4A•AQ4N exhibited negligible cytotoxicity to 3T3 cells at concentrations ranging from 0 to 200 × 10^−6^ m, indicating the low side effect of CAC4A•AQ4N on normal cells. As showed in Figure S27b (Supporting Information), no obvious hemolysis was observed after incubation red blood cells with CAC4A, AQ4N and CAC4A•AQ4N, indicating the ignorable damage of CAC4A•AQ4N to red cells. As shown in Figure S28 (Supporting Information), no appreciable changes of the body weight in all experimental groups were observed, suggesting the insignificant systemic toxicity induced by CAC4A•AQ4N. Furthermore, major organs of mice after treatment were collected and stained by H&E for histopathologic analysis (Figure S29, Supporting Information). Neither apparent histopathological abnormalities nor inflammation was investigated, corresponding to the low side effects of CAC4A•AQ4N. In addition, there was no obvious variation for the important parameters in the blood biochemistry and hematology analyses (Figure S30, Supporting Information), further proving that CAC4A•AQ4N have a great biological safety for cancer radiotherapy.

Macrocycles exhibits an ability to accommodate different drugs as would be expected for a putative universal platform of supramolecular prodrugs based on host–guest interactions. To verify the universality of the hypoxia‐responsive macrocycle for supramolecular radiotherapy, a series of radiosensitive small molecule drugs that are widely applied in clinic treatment, such as paclitaxel (PTX), docetaxel (DOC), doxorubicin (DOX), pirarubicin (THP), camptothecin (CPT), irinotecan (CPT‐11), hydroxycamptothecin (HCPT), topotecan (TPT), tamoxifen (TAM), hydroxychloroquine (HCQ), and lovastatin (LOV), were employed to detect their binding affinities with CAC4A. As expected, the association constants between CAC4A and these drugs were all higher than 10^5^ M^−1^ (Table S2 and Figures S31 and S32, Supporting Information). The high binding affinity is a vital requirement for drug delivery, because supramolecular complexes with low binding affinities may suffer unexpected drug release in physiological environment. Moreover, the hypoxia response of CAC4A was further confirmed by incubating cancer cells with the complexation of CAC4A with different radiosensitive small molecule drugs (e.g., DOX and THP) under normoxia and hypoxia conditions (Figure S33, Supporting Information). Incubation of cancer cells with supramolecular complexes under hypoxia exhibited remarkable stronger fluorescence signals of drugs than those under normoxia. The increased fluorescence manifested the outstanding hypoxia responsive drug release performance of the supramolecular complexes, which could be attributed to the removal of hypoxia sensitive azo groups in CAC4A. Moreover, as shown in Figure S34 (Supporting Information), the remarkable stronger fluorescence signals of hypoxic cells incubated with supramolecular complexes than that of free drugs, suggested that CAC4A was able to facilitate the delivery of drugs and increase their accumulation in cancer cells. Furthermore, we surveyed the radiosensitizing efficacy of supramolecular complexes consisting of CAC4A and different radiosensitive small molecule drugs (e.g., DOX, THP, TPT, and TAM) by calculating SER values based on the clonogenic assay (Figure S35, Supporting Information). As shown in Table S3 (Supporting Information), the SER value of supramolecular complex against 4T1 cells under hypoxia condition (CAC4A•DOX: 2.62; CAC4A•THP:2.31; CAC4A•TPT: 1.92; CAC4A•TAM: 1.89) was much higher than that of the corresponding free drugs (DOX: 1.62; THP:1.33; TPT: 1.29; TAM: 1.20), which suggested that complexation with CAC4A increased the radio‐therapeutic effect of radiosensitive small molecule drugs significantly. As a result, CAC4A acts potentially as carrier for these radiosensitizing drugs. A general platform for supramolecular radiotherapy is envisaged.

## 3 Conclusion

In conclusion, we successfully developed a supramolecular radiotherapy strategy based on hypoxia‐responsive macrocycle to achieve targeted radiated cancer suppression. Through complexing radiosensitive drugs within macrocyclic hosts to form a supramolecular formulation, our strategy could remarkably strengthen the radiation‐caused cancer cell proliferation inhibition, aggravate the radiation‐induced DNA damage and promote the apoptosis of irradiated cells. In vivo animal experiments demonstrated that supramolecular radiotherapy could not only achieve more significant therapeutic efficacy over free radiosensitive drugs, but also reduce the drug dosage and radiation dose to minimize undesirable damage to normal tissues. The enhanced radiotherapeutic efficiency of our strategy was mainly attributed to the improved tumor accumulation and cellular internalization of drug by delicate host–guest complexation. In our present study CAC4A•AQ4N serves as a proof‐of‐concept example of supramolecular radiotherapy, this strategy and the corresponding conclusions are reasonably transferable to other radiosensitive drugs for establishing a universal platform as the hypoxia‐responsive macrocycle is able to accommodate a series of drugs with high affinities. Therefore, this work offers a new horizon for realizing safe and reinforced cancer radiotherapy in a simple way.

## Conflict of Interest

The authors declare no conflict of interest.

## Supporting information

Supporting Information

## Data Availability

Research data are not shared.
