# Supplementary material for: Supramolecular Radiosensitizer Based on Hypoxia‐Responsive Macrocycle
Source: Adv Sci (Weinh). 2022 Jan 7;9(6):2104349. doi: 10.1002/advs.202104349 (PMC8867162; doi:10.1002/advs.202104349)
Supplement: Supplementary file 1 — Supporting Information [file ADVS-9-2104349-s001.pdf]

ADVANCED SCIENCE
Open Access

Supporting Information

for *Adv. Sci.,* DOI: 10.1002/advs.202104349

Supramolecular Radiosensitizer Based on Hypoxia-Responsive Macrocycle

Xiaoxue Hou, Yu-Xuan Chang, Yu-Xin Yue, Ze-Han Wang, Fei Ding, Zhi-Hao Li, Hua-Bin Li, Yicheng Xu, Xianglei Kong, Fan Huang, Dong-Sheng Guo* and Jianfeng Liu**

Supporting Information

Supramolecular Radiosensitizer Based on Hypoxia-Responsive Macrocycle

Xiaoxue Hou, Yu-Xuan Chang, Yu-Xin Yue, Ze-Han Wang, Fei Ding, Zhi-Hao Li, Hua-Bin Li, Yicheng Xu, Xianglei Kong, Fan Huang, * *Dong-Sheng Guo* * *and Jianfeng Liu* *

Table of Contents

1. Experimental Procedures 4

1.1 Materials 4

1.2 Apparatus 4

1.3 Measurement of binding affinities between various calixarenes and guests 5

1.4 Theoretical calculation for the simulation of CAC4A•AQ4N binding geometry 6

1.5 *In vitro* simulation of hypoxia response of CAC4A•AQ4N 6

1.6 Determination of encapsulation percentage (EN%) of AQ4N@DPPC nanoparticles and CAC4A•AQ4N 6

1.7 Fluorescence responses of CAC4A•AQ4N upon addition of various biologically coexisting species 7

1.8 Cellular uptake 7

1.9 *In vitro* sensitization efficiency study 8

1.10 Mice 9

1.11 Biodistribution of CAC4A•AQ4N. 10

1.12 *In Vivo* Antitumor Experiments 10

1.13 Safety evaluation of CAC4A•AQ4N.. 12

1.14 Statistical analysis.. 13

2. Results and Discussion 15

2.1 Syntheses of CAC4A, CAC5A, CAC6A, CAC8A and NH_2C4A 15

2.2 Fluorescence titrations 21

2.3 UV-Vis spectra of CAC4A, AQ4N and CAC4A•AQ4N 25

2.4 Job's plot for the complexation of CAC4A•AQ4N 25

2.5 The mechanism behind the strong binding between CAC4A and AQ4N 25

2.6 UV-Vis spectra of AQ4N when reduced by various concentrations of SDT 27

2.7 The integrity of CAC4A, AQ4N and CAC4A•AQ4N in heme 28
2.8 Hypoxic response of CAC4A•AQ4N 29
2.9 Cellular uptake of CAC4A •AQ4N 30
2.10 The cytotoxcity of CAC4A •AQ4N 30
2.11 *In vitro* sensitization efficiency study 31
2.12 Biodistribution of CAC4A•AQ4N 34
2.13 TUNEL staining of tumor slices from mice treated with CAC4A•AQ4N 34
2.14 Safety evaluation of CAC4A•AQ4N 35
2.15 Binding data of CAC4A with various drug molecules 38
2.16 Hypoxic response of CAC4A•DOX and CAC4A•THP 38
2.17 Cellular uptake of CAC4A•DOX and CAC4A•THP 38
2.18 The radiosensitizing efficacy of supramolecular complexes 40
3. References 41

1. Experimental Procedures

1.1 Materials

All the reagents and solvents were commercially available and used as received unless otherwise specified purification. Concentrated hydrochloric acid (37%) was purchased from Tianjin Jindongtianzheng Precision Chemical Reagent Factory. Sodium hydroxide was purchased from Fuchen Chemicals. Arginine and banoxantrone dihydrochloride (AQ4N) were purchased from Macklin. PBS buffer (10 mM, pH = 7.4) was purchased from Shanghai Yuanye Bio-Technology. Lovastatin was purchased from 3A chemicals. Creatinine and hydroxychloroquine were purchased from J&K Chemical. 1,2-dipalmitoyl-sn-glycero-3-phosphocholine (DPPC) was purchased from Avanti. 4-aminobenzoic acid, sodium nitrite and urea were purchased from Aladdin. Nicotinamide adenine dinucleotide (NAD) was purchased from TCI. Adenosine triphosphate (ATP) was obtained from Solarbio. Glutathione and valine were obtained from Adamas Reagent. Alanine, glutamine, glucose and proline were purchased from Heowns. Lysine and sodium dithionite (SDT) were obtained from Meryer. Glycine was purchased from MCE. $MgCl_2$ was obtained from Energy Chemical. NaCl, KCl and $CaCl_2$ were purchased from Guangfu Fine Chemical Research Institute. Crystal violet, 4’6-diamidino-2-phenylindole (DAPI), Hematoxylin-Eosin/H&E staining kit and terminal deoxynucleotidyl transferase dUTP nick end labeling (TUNEL) kit was obtained from Solarbio. All antibodies for immunofluorescence staining were purchased from Abcam and apoptosis kit was purchased from BD company. The mice were purchased from Beijing Vital River Laboratory Animal Corporation (Beijing, China).

1.2 Apparatus

^{1}H and ^{13}C NMR data were recorded on a Bruker AV400 spectrometer. Mass spectra were performed on a Varian 7.0T FTICR-MS (MALDI-TOF). Steady-state and kinetic fluorescence spectra were recorded in a conventional quartz cell (light path 10 mm) on a Cary Eclipse fluorescence spectrophotometer equipped with a Cary single-cell peltier accessory.

UV-Vis spectra were recorded in a conventional quartz cell (light path 10 mm) on a Cary 100 UV-Vis spectrophotometer equipped with a Cary dual-cell peltier accessory. Fluorescence microscopy images were observed by an inverted fluorescence microscope (Leica, DMI 6000B).

1.3 Measurement of binding affinities between various calixarenes and guests

Direct fluorescence titrations. The complexation of calixarenes (CAC4A, CAC5A, CAC6A, CAC8A, NH_2C4A) and AQ4N were obtained through direct fluorescence titrations. Briefly, a mixed solution containing known amount of calixarene and AQ4N was sequentially injected into 2.50 mL AQ4N solution in a quartz cuvette. The AQ4N concentration in mixed solution and cuvette is the same to keep AQ4N concentration constant in the course of titrations. The fluorescence intensity was measured (λ_{ex} = 610 nm, λ_{ex} = 685 nm) before the first addition and after every addition until a plateau was reached. The fluorescence intensities were fitted according to 1:1 binding stoichiometry based the concentration of AQ4N.

Competitive fluorescence titrations. The complexation of CAC4A and hydroxychloroquine, lovastatin were obtained through competitive fluorescence titrations using Rhodamine B (RhB) as reporter dye.[1] Briefly, a mixed solution containing known amounts of CAC4A, AQ4N, and a competitive guest (hydroxychloroquine or lovastatin) was sequentially injected into 2.50 mL CAC4A and AQ4N solution in a quartz cuvette. The CAC4A and AQ4N concentrations in mixed solution and cuvette is the same to keep CAC4A and AQ4N concentrations constant in the course of titrations. The fluorescence intensity was measured (λ_{ex} = 610 nm, λ_{ex} = 685 nm) before the first addition and after every addition until a plateau was reached. The fluorescence intensities were fitted according to 1:1 binding stoichiometry based the concentration of the guest.

All fluorescence titrations were measured in PBS buffer (10 mM, pH = 7.4) at 25 °C. The fitting functions can be downloaded from the website of Prof. Nau's group (http://www.jacobs-university.de/ses/wnau).

1.4 Theoretical calculation for the simulation of CAC4A•AQ4N binding geometry

The assembly of CAC4A•AQ4N was solvated in an equilibrated box of water. The overall charge neutrality was achieved by adding five Na^+ ions to the solution. The system (cubic box ~ 45 × 45 × 45 $Å^3$, total 8177 atoms) before production simulation underwent i) 5000 steps minimization and 100 ps MD simulation with host and guest restrained, and ii) 2 ns of water equilibration without restrained. Then, their interactions were investigated by performing a 100 ns MD simulation. The scalable program NAMD 2.14[2] with the CHARMM36 force field,[3] and the TIP3P water model[4] were used to perform the MD simulations. Visualization and analysis of all the MD trajectories were carried out with the VMD program.[5]

1.5 *In vitro* simulation of hypoxia response of CAC4A•AQ4N

Drug release simulation. To simulate the hypoxia-triggered drug release of CAC4A•AQ4N *in vitro*, SDT was added to CAC4A•AQ4N (30.00/10.00 μM) to reach five target concentrations (0, 0.50, 1.00, 1.50 and 2.50 mM) in PBS buffer (10 mM, pH = 7.4) at 25 °C, and the fluorescence spectra of AQ4N were recorded, λ_{ex} = 517 nm.

Release kinetics simulation. To simulate the release kinetics of CAC4A•AQ4N in hypoxia environment *in vitro*, SDT was added to CAC4A•AQ4N (30.00/10.00 μM) to reach the target concentration of 1.00 mM in PBS buffer (10 mM, pH = 7.4) at 37 °C. The fluorescence intensity at 590 nm was monitored in real time, λ_{ex} = 517 nm.

1.6 Determination of encapsulation percentage (EN%) of AQ4N@DPPC liposome and CAC4A•AQ4N

The AQ4N@DPPC liposome was prepared according to standard protocol.[6] Briefly, 0.50 mmol DPPC was dissolved in chloroform and dried on a rotatory evaporator. The formed

lipid film was then hydrated with 1.00 mL 0.50 mM AQ4N in PBS buffer (10 mM, pH = 7.4) at 45 °C. The obtained liposome was then extruded through a 200 nm polycarbonate filter at 45 °C for 20 times, and the unencapsulated AQ4N was removed *via* a sephadex G-25 column. The concentration of encapsulated AQ4N was quantified using its absorbance at 610 nm with mass extinction coefficient of 22.5 mL mg^{-1} cm^{-1}; The CAC4A•AQ4N was prepared by mixing the PBS buffer (10 mM, pH = 7.4) solutions of CAC4A and AQ4N together to reach the final concentrations of 0.50 mM, respectively. The concentration of encapsulated AQ4N was calculated by the association constant of $(1.07 \pm 0.11) \times 10^6$ M^{-1}.

The EN% of AQ4N was calculated as follow:

encapsulation efficiency (%) = $(m_{AQ4N\text{-}loaded}/m_{AQ4N}) \times 100\%$ (1)

$m_{AQ4N\text{-}loaded}$ is the mass of AQ4N encapsulated in DPPC or CAC4A, m_{AQ4N} is the mass of total AQ4N.

1.7 Fluorescence responses of CAC4A•AQ4N upon addition of various biologically coexisting species

Various biological coexisting species of blood were added to CAC4A•AQ4N (10.00/10.00 μM) in PBS buffer (10 mM, pH = 7.4) at 25 °C and stirred for 30 min to monitor the fluorescence intensity of AQ4N. The fluorescence of AQ4N alone were used as control. The biological coexisting species and their concentrations used in these experiments were as follows: NAD 24.00 μM, BSA 10.00 μg mL^{-1}, glutathione 8.00 μM, urea 4.00 mM, ATP 0.40 μM, glucose 5.00 mM, creatinine 80.00 μM, glutamine 0.50 mM, alanine 0.40 mM, glycine 0.30 mM, arginine 0.14 mM, valine 0.20 mM, lysine 0.20 mM, proline 0.20 mM, K^+ 4.50 mM, Ca^{2+} 2.50 mM, Na^+ 144.00 mM and Mg^{2+} 0.80 mM. The concentrations of all above components refer to their concentrations in human blood.[7]

1.8 Cellular uptake

Murine breast cancer 4T1 cells were cultured in Roswell Park Memorial Institute 1640 (RPMI 1640) medium with 10% fetal bovine serum (FBS) and 1% penicillin/streptomycin. All cells were seeded in culture dish under a humidified environment containing 5% CO_2 at 37 °C.

The 4T1 cells were plated in confocal microscopic dishes at a density of 2×10^5 cells per dish for incubation of 24 h. The cells were then incubated with fresh RPMI-1640 medium containing free drugs (AQ4N, DOX and THP) and the complexes (CAC4A•AQ4N, CAC4A•DOX and CAC4A•THP) with an equivalent drug concentration of 10 μM, followed by placed in normoxia condition (a humidified environment containing 5% CO_2) or hypoxia condition (94% N_2, 5% CO_2, 1% O_2). After incubation of various times (1, 3, 6 and 24 h), the cells were wash with PBS three times, immobilized with 4% paraformaldehyde solution for 30 min, stained with nuclear dye DAPI for 15 min and supplement 1 mL fresh PBS. The fluorescence images were recorded by an inverted fluorescence microscope (Leica, DMI6000B).

To further investigate the endocytosis mechanisms of CAC4A•AQ4N, 4T1 cells were treated with fresh complete culture medium containing the following endocytosis inhibitors for inhibition of energy-dependent endocytosis: NaN_3 (10 mM) and low temperature (4 °C). After 30 min, the medium was replaced with fresh complete medium containing CAC4A•AQ4N/AQ4N (AQ4N concentration: 10 μM) and same kind of endocytosis inhibitor for another 2 h. Control cells were treated with complete culture medium. After that, the medium was removed and the cells were washed with PBS several times before flow cytometry analysis.

1.9 *In vitro* sensitization efficiency study

Clonogenic survival assay. Briefly, 4T1 cells were plated in 6-well plates at a density of 1,000 per well in a humidified 5% CO_2 environment at 37 °C for 24 h. The cells were then treated with PBS, CAC4A (5 μM), AQ4N (1 μM) and CAC4A•AQ4N (5/1 μM) for another

24 h. The cells cultured in fresh medium without additional processing were used as the control. Afterwards, the cells were exposed with γ-ray irradiation of different dosages (0, 2, 4 and 6 Gy) and further incubated for another 7 days. The forming colonies were stained with 0.25% crystal violet. The surviving fraction and sensitizer enhancement ratio (SER) were calculated according to the standard methods.[8] The SER values of CAC4A•DOX, CAC4A•THP, CAC4A•TPT and CAC4A•TAM were also calculated according to the above procedures.

Immunofluorescence staining of γ-H2AX. The 4T1 cells were plated in confocal microscopic dishes at a density of 2×10^5 cells per dish for incubation of 24 h. After treatment with PBS, CAC4A (10 μM), AQ4N (10 μM) and CAC4A•AQ4N (10/10 μM) for 24 h, the cells were then irradiated with γ-ray at the dosage of 6 Gy. Following a total of 2 h incubation at 37 °C, the cells were washed with PBS three times, immobilized with 4% paraformaldehyde solution for 30 min, permeabilized with pre-cooled methanol glycol solution for 15 min at -20 °C, blocked by 1% BSA solution for 1 h at room temperature. Subsequently, the cells were incubated with anti-phospho-histone γ-H2AX mouse monoclonal antibody overnight at 4 °C followed by PBS washing and being probing with horseradish peroxidase-conjugated anti-mouse secondary antibodies for 1 h at room temperature. The cell nuclei were then stained with DAPI for 15 min. Finally, the fluorescence signals of γ-H2AX were detected by an inverted fluorescence microscope (Leica, DMI6000B).

Cell apoptosis analysis. The inducing apoptosis performance was evaluated by flow cytometry using the Annexin V-FITC/PI apoptosis detection kit. Briefly, 4T1 cells were treated with PBS, CAC4A (10 μM), AQ4N (10 μM) and CAC4A•AQ4N (10/10 μM) for 24 h after cell attachment and subjected to 6 Gy of γ-ray irradiation. After another 24 h incubation at 37 °C, the cells were treated based on the instructions of the apoptosis detection kit and subsequently analyzed by flow cytometry.

1.10 Mice

For animals and tumor model, female BALB/c mice at 6-8 weeks and female nude BALB/c nude mice at 5-6 weeks were purchased from Vital River Laboratory Animal Technology (Beijing, China). All animal procedures were performed in accordance with the Guidelines for the Care and Use of Laboratory Animals of Peking Union Medical College and experiments were approved by the Animal Experiments and Ethics Review Committee of the Institute of Radiation Medicine, Chinese Academy of Medical Sciences.

1.11 Biodistribution of CAC4A•AQ4N

BALB/c nude mice at 5-6 weeks were injected subcutaneously 1×10^6 4T1 cancer cells into the right chest. After establishing the xenograft 4T1 tumor-bearing mouse model, the mice with tumor volumes at around 200 mm^3 were randomized into 2 groups and intravenous injected with 100 μL of AQ4N (250 μM, 0.63 mg kg^{-1}) and CAC4A•AQ4N (250 μM for AQ4N, 0.63 mg kg^{-1}). The *in vivo* and *ex vivo* distribution of AQ4N and CAC4A•AQ4N were imaged by IVIS Lumina imaging system (Caliper Life Sciences, USA) at the time of 1, 6, 12, 24, 48 and 72 h after injection, respectively. Fluorescence images were analyzed using Living Image 3.1 (Caliper Life Sciences). The quantitative data were obtained by normalizing the parameters of fluorescence images.

AQ4N is a hypoxia-activated prodrug and forms its reduction product AQ4 under hypoxic condition. It is essential to investigate the fluorescence changes during the process of AQ4N-to-AQ4 conversion. Therefore, we detected the fluorescence spectra of AQ4N and AQ4. As shown in Figure S1a, the fluorescence spectra of AQ4N and AQ4 were virtually overlapped at the same concentration (20 μM) and exhibited maximum fluorescence intensities at 700 nm. We then evaluated the fluorescence intensities at 700 nm of AQ4N and AQ4 after incubation with homogenate of major organs (heart, liver, spleen, lung, kidney) and tumor. As shown in Figure S1b, there was negligible differences in fluorescence intensities at

700 nm of AQ4N and AQ4, suggesting the inapparent effect on the fluorescence changes of AQ4N-to-AQ4 conversion in the distribution and metabolism process. Furthermore, we assessed the fluorescence intensities at 700 nm of AQ4N and AQ4 with different concentrations (1.25, 5, 10, 15 and 20 μM) before and after incubation with tumor homogenate. As shown in Figure S1c and 1d, the fluorescence intensities at 700 nm of AQ4N and AQ4 exhibited similar linear enhancement with the concentrations. According to the above results, it is reasonable to conclude that the distribution and metabolism could be measured by fluorescence quantification, which was attributed to the negligible fluorescence changes of AQ4N under normoxic and hypoxic conditions.

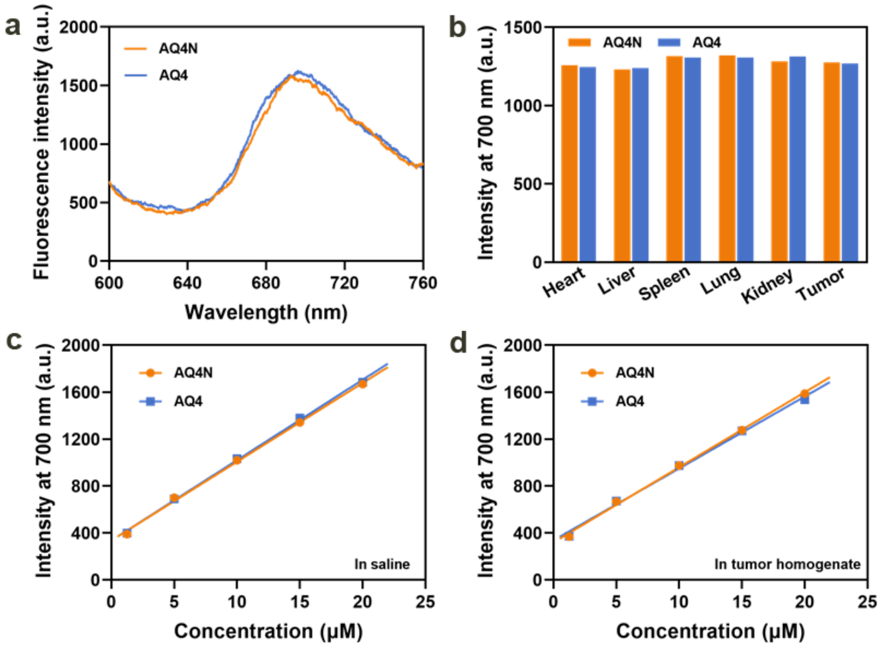

Figure S1. a) Fluorescence spectra of AQ4N and AQ4 at the same concentration (20 μM). b) Fluorescence intensities of AQ4N and AQ4 at 700 nm after incubation with homogenate of major organs (heart, liver, spleen, lung, kidney) and tumor. c) The intensity curves of AQ4N and AQ4 at concentrations ranging from 1.25 to 20 μM. d) The intensity curves of AQ4N and AQ4 at concentrations ranging from 1.25 to 20 μM after incubation with tumor homogenate.

1.12 *In Vivo* Antitumor Experiments

Antitumor efficiency of CAC4A•AQ4N. To assess the antitumor ability of CAC4A•AQ4N, 1.5×10^6 4T1 cancer cells were injected subcutaneously into the right flanks of the mice. The mice with tumor volumes at around 50 mm^3 were randomized into 10 groups (5 mice per group) and intravenous injected with PBS (100 μL per dose), CAC4A (250 μM, 100 μL per dose, 1.27 mg kg^{-1}), AQ4N (250 μM, 100 μL per dose, 0.63 mg kg^{-1}), AQ4N (2 mM, 100 μL per dose, 5 mg kg^{-1}) and CAC4A•AQ4N (250 μM for AQ4N, 100 μL per dose, 0.63 mg kg^{-1}) with/without receiving γ-ray radiation at 24 h post-injection and simultaneous monitoring the tumor volumes every two days over 21 days. Tumors were measured by using a vernier calipers and the volume (V) was calculated to be $V = d^2 \times D/2$, where d is the shortest and the D is longest diameter of the tumor in mm respectively. To assess potential toxicities, mice were monitored for weight loss. Tumors were collected for H&E analysis, terminal deoxynucleotidyl transferase dUTP nick end labeling (TUNEL) assay and immunofluorescence staining.

Histological analysis. Tumors were immobilized with 4% paraformaldehyde solution, embedded in paraffin and sliced 6 μm thick sections. The H&E analysis, TUNEL assay and immunofluorescence staining were conducted in accordance with the standard protocols of corresponding assay kit. The histological analysis was investigated by an optical microscope (Leica, DMI6000B).

1.13 Safety evaluation of CAC4A•AQ4N

Cell Cytotoxicity Study. 4T1 cells were plated in 96-well plates at a density of 5×10^3 cells per well. After incubation of 24 h in a humidified 5% CO_2 environment, cells were cultured in fresh medium containing with different concentrations of CAC4A, AQ4N and CAC4A•AQ4N. Cells cultured in fresh medium without drug were used as the control. Following a total of 24 h incubation at 37°C, 20 μL of MTT solution (5 mg/mL) was added into each well. After incubation for another 4 h, the medium was removed and 150 μL of

DMSO was added to dissolve the purple formazan. The optical absorption at 570 nm was immediately measured by a Varioskan Flash (Thermo Scientific Company, USA).

Hemolysis assay. For hemolysis assay, fresh rat blood cells were washed three times with sterile PBS buffer and harvested by centrifugation at 3000 rpm for 5 min. Then, 0.4 mL of diluted rat red blood cell suspension was mixed with 400 μL CAC4A, AQ4N and CAC4A•AQ4N at predetermined concentration in 1.5 mL Eppendorf tube at 37 ℃ for 4 h. The final solutions were centrifuged (3000 rpm, 5min) and ultraviolet absorptive value (570 nm) of resultant supernatant (100 μL) was measured by a microplate reader. Rat red blood cell suspension mixed with PBS buffer was used as a negative control, while rat red blood cell suspension mixed with Triton X-100 (0.1%) solution was used as a positive control. The hemolysis ratio (%) = (absorbance of experimental sample - absorbance of negative control sample)/(absorbance of positive control sample - absorbance of negative control sample) ×100.

Blood routine and blood biochemical examination. To evaluate the clinical potential of CAC4A•AQ4N, BALB/c mice were randomly divided into 4 groups (3 mice per group), followed by intravenous administration with PBS (100 μL per dose), AQ4N (250 μM, 100 μL per dose, 0.63 mg kg^{-1}), CAC4A (250 μM, 100 μL per dose, 1.27 mg kg^{-1}) and CAC4A•AQ4N (250 μM for AQ4N, 100 μL per dose, 0.63 mg kg^{-1}) three times at the interval of one week. Blood samples were then collected for blood chemistry assay and blood routine assay on Day 21.

Histopathologic analysis. Major organs (heart, liver, spleen, lung and kidney) were immobilized with 4% paraformaldehyde solution, embedded in paraffin and sliced 6 μm thick sections. The sections of major organs were collected and stained with hematoxylin-eosin (H&E) for histopathologic analysis.

1.14 Statistical analysis

All data were shown mean ± standard deviation (s.d.) of three independent experiments unless otherwise stated. Statistical analysis was determined based on the student's *t*-test and ANOVA analysis through the software of SPSS 25.0, and *P*-value thresholds of < 0.05 (*), < 0.01 (**), or < 0.001 (***) were considered significant.

2. Results and Discussion

2.1 Syntheses of CAC4A, CAC5A, CAC6A, CAC8A and NH_2C4A

Syntheses of CAC4A, CAC5A, CAC6A, CAC8A

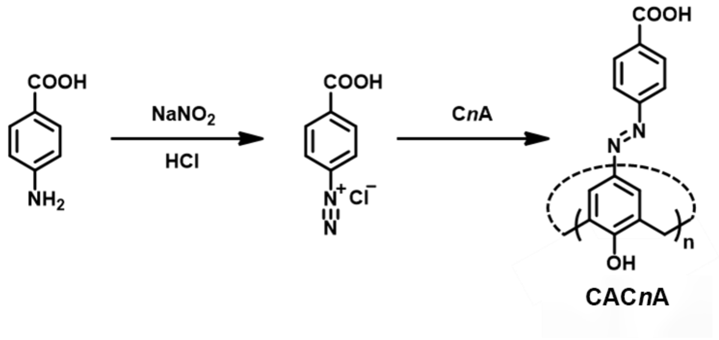

Scheme S1. The synthetic route of CAC*n*As (n = 4, 5, 6, 8).

5,11,17,23-tetrakis[(p-carboxyphenyl)azo]-25,26,27,28-tetrahydroxycalix[4]arene (CAC4A).[9] 4-aminobenzoic acid (1.37 g, 10 mmol) and concentrated hydrochloric acid (2 mL, 37%) were dissolved in 15 mL water in a round bottom flask. After cooling the solution to 2 °C under an ice-water bath, a solution of sodium nitrite (10 mL, 10 mmol, in water) was slowly added, while the temperature was controlled to be lower than 5 °C. The obtained solution was slowly added into a solution of 25,26,27,28-tetrahydroxycalix[4]arene (C4A, 1.00 g, 2.36 mmol) and sodium acetate (2.46 g, 30 mmol) in MeOH-DMF (26 mL, 5:8, v:v) to obtain a red suspension. After stirring at room temperature for 2 h, hydrochloric acid solution (37%) was added until the solution was adjusted to pH = 1. After warming to 60 °C for 30 min, the mixture was filtered and washed with water and MeOH and dried under vacuum. CAC4A was obtained as black solid in a quantitative yield. ^{1}H NMR (400 MHz, DMSO-d_6), δ 8.02 (d, 8H, J = 8.6 Hz, Ar-H), 7.81 (d, 8H, J = 8.6 Hz, Ar-H), 7.80 (s, 8H, calix-Ar-H), 4.37 and 3.72 (s, 8H, Ar-CH_2-Ar) ppm.

5,11,17,23,29-pentakis[(p-carboxyphenyl)azo]-31,32,33,34,35-pentahydroxycalix[5]arene (CAC5A). CAC5A was prepared according to the method similar to that of CAC4A and was obtained as black solid in a quantitative yield. ^{1}H NMR (400 MHz, Pyridine-d_5), δ 12.22 (s,

10H, Ar-OH), 8.54 (d, 10H, *J* = 8.3 Hz, Ar-H), 8.24 (s, 10H, calix-Ar-H), 8.06 (d, 10H, *J* = 8.2 Hz, Ar-H), 4.41 (s, 10H, Ar-CH_2-Ar) ppm; ^{13}C NMR (100 MHz, Pyridine-d_5), δ 168.95, 161.78, 156.06, 146.00, 133.56, 131.59, 130.23, 125.70, 122.90, 33.41 ppm; Mass spectrum (MALDI-TOF): $[M-H]^-$: *m/z* calcd. for ($C_{70}H_{49}N_{10}O_{15}^-$): 1270.220, found: 1269.922.

5,11,17,23,29,35-hexakis[(p-carboxyphenyl)azo]-37,38,39,40,41,42-hexahydroxycalix[6]arene (CAC6A). CAC6A was prepared according to the method similar to that of CAC4A, and was further purified through recrystallization using ethyl acetate-DMF. The obtained crimson solid was further dissolved in 1 M NaOH and stirred at 60 °C for 30 min. The red suspension was filtered, and the filtrate was acidified with concentrated hydrochloric acid (37%) and stirred at 60 °C for 30 min. The resulting suspension was filtered, washed with ethanol, and dried under vaccum to obtain the black solid of CAC6A in a 48% yield. ^{1}H NMR (400 MHz, DMSO-d_6), δ 8.05 (d, 12H, *J* = 8.2 Hz, Ar-H), 7.85 (d, 12H, *J* = 8.6 Hz, Ar-H), 7.82 (s, 12H, calix-Ar-H), 4.00 (s, 12H, Ar-CH_2-Ar) ppm; ^{13}C NMR (100 MHz, Pyridine-d_5), δ 169.03, 162.09, 156.30, 145.84, 133.32, 131.52, 130.57, 125.58, 122.90, 34.67 ppm; Mass spectrum (MALDI-TOF): $[M-H]^-$: *m/z* calcd. for ($C_{84}H_{59}N_{12}O_{18}^-$): 1524.466, found: 1524.383.

5,11,17,23,29,35,41,47-octakis[(p-carboxyphenyl)azo]-49,50,51,52,53,54,55,56-octahydroxycalix[8]arene (CAC8A). CAC8A was prepared according to the method similar to that of CAC6A and was obtained as black solid in a 11% yield. ^{1}H NMR (400 MHz, DMSO-d_6), δ 8.03 (d, 16H, *J* = 8.6 Hz, Ar-H), 7.79 (d, 16H, *J* = 8.6 Hz, Ar-H), 7.73 (s, 16H, calix-Ar-H), 4.05 (s, 16H, Ar-CH_2-Ar) ppm; ^{13}C NMR (100 MHz, Pyridine-d_5 and DMSO-d_6, 1:1, v:v), δ 167.95, 161.62, 155.67, 145.25, 132.25, 131.01, 130.14, 124.97, 122.53, 33.24; Mass spectrum (MALDI-TOF): $[M+Na]^+$: *m/z* calcd. for ($C_{112}H_{80}N_{16}O_{24}Na^+$): 2056.545, found: 2056.627.

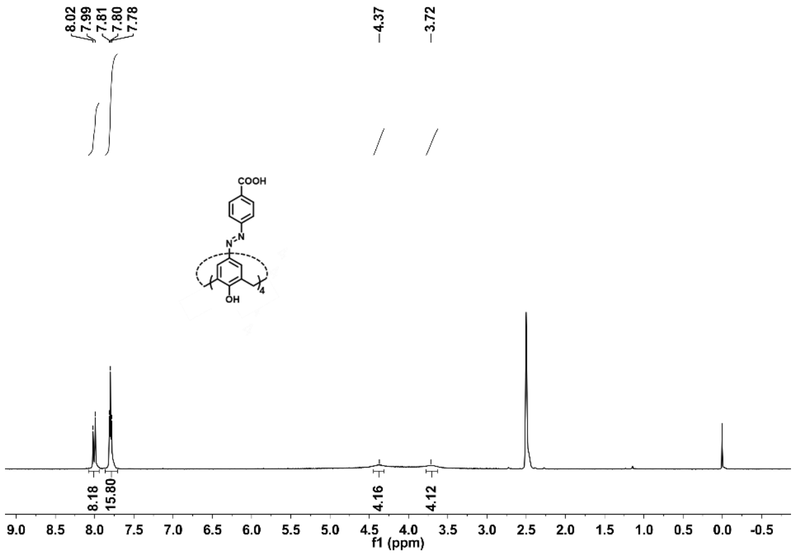

Figure S2. ^{1}H NMR spectrum of CAC4A in DMSO-d_6, 400 MHz, 25 °C.

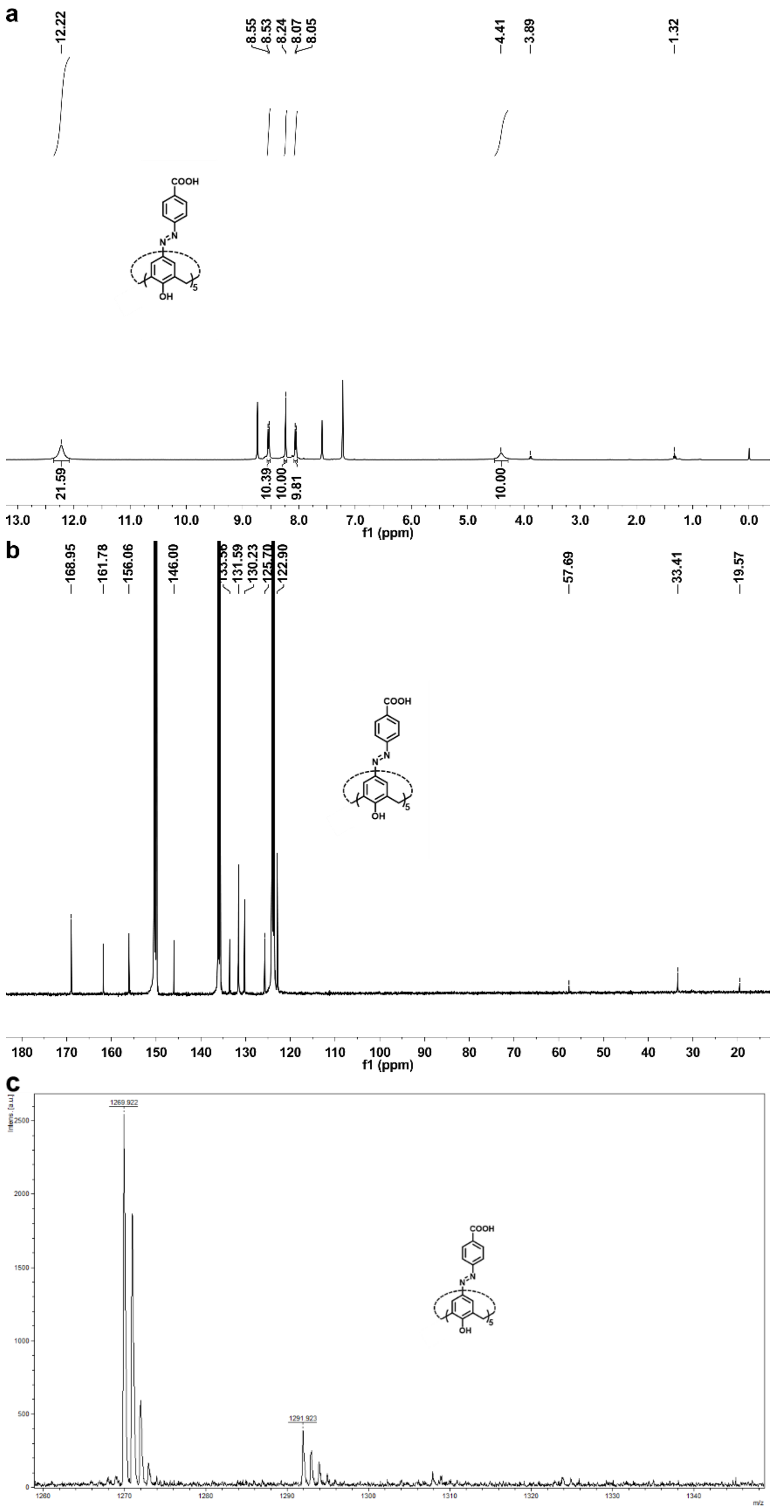

Figure S3. a) ^{1}H NMR spectrum of CAC5A in Pyridine-d_5, 400 MHz, 25 °C. Two signals (3.89 and 1.32 ppm) for ethanol were observed, probably because ethanol was encapsulated into the cavity of CAC5A. b) ^{13}C NMR spectrum of CAC5A in Pyridine-d_5, 100MHz, 25 °C. Two signals (57.69 and 19.57 ppm) for ethanol were observed. c) Mass spectrum (MALDI-TOF) of CAC5A.

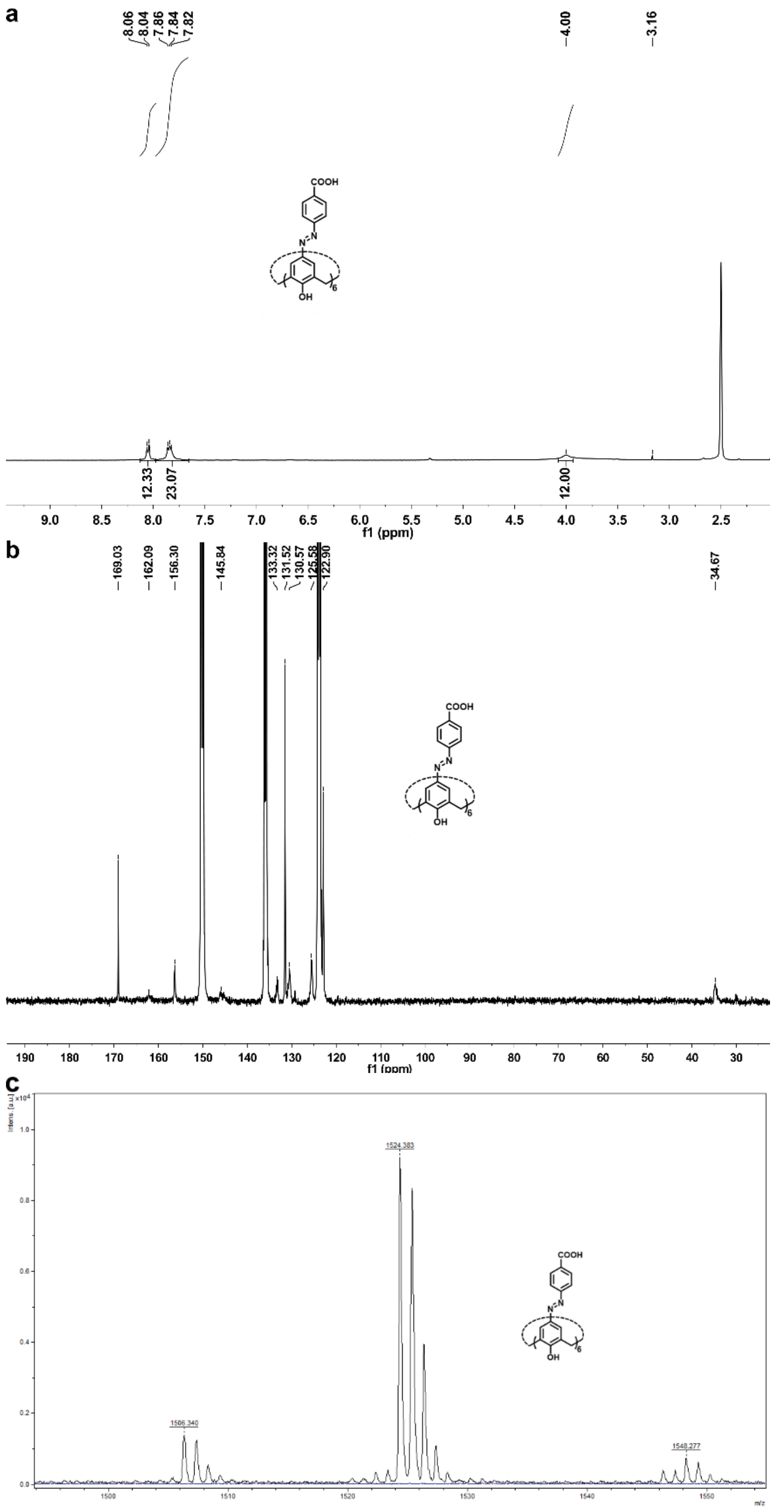

Figure S4. a) ^{1}H NMR spectrum of CAC6A in DMSO-d_6, 400 MHz, 25 °C. A signal (3.16 ppm) for methanol was observed, probably because methanol was encapsulated into the cavity of CAC6A. b) ^{13}C NMR spectrum of CAC6A in Pyridine-d_5, 100 MHz, 25 °C. c) Mass spectrum (MALDI-TOF) of CAC6A.

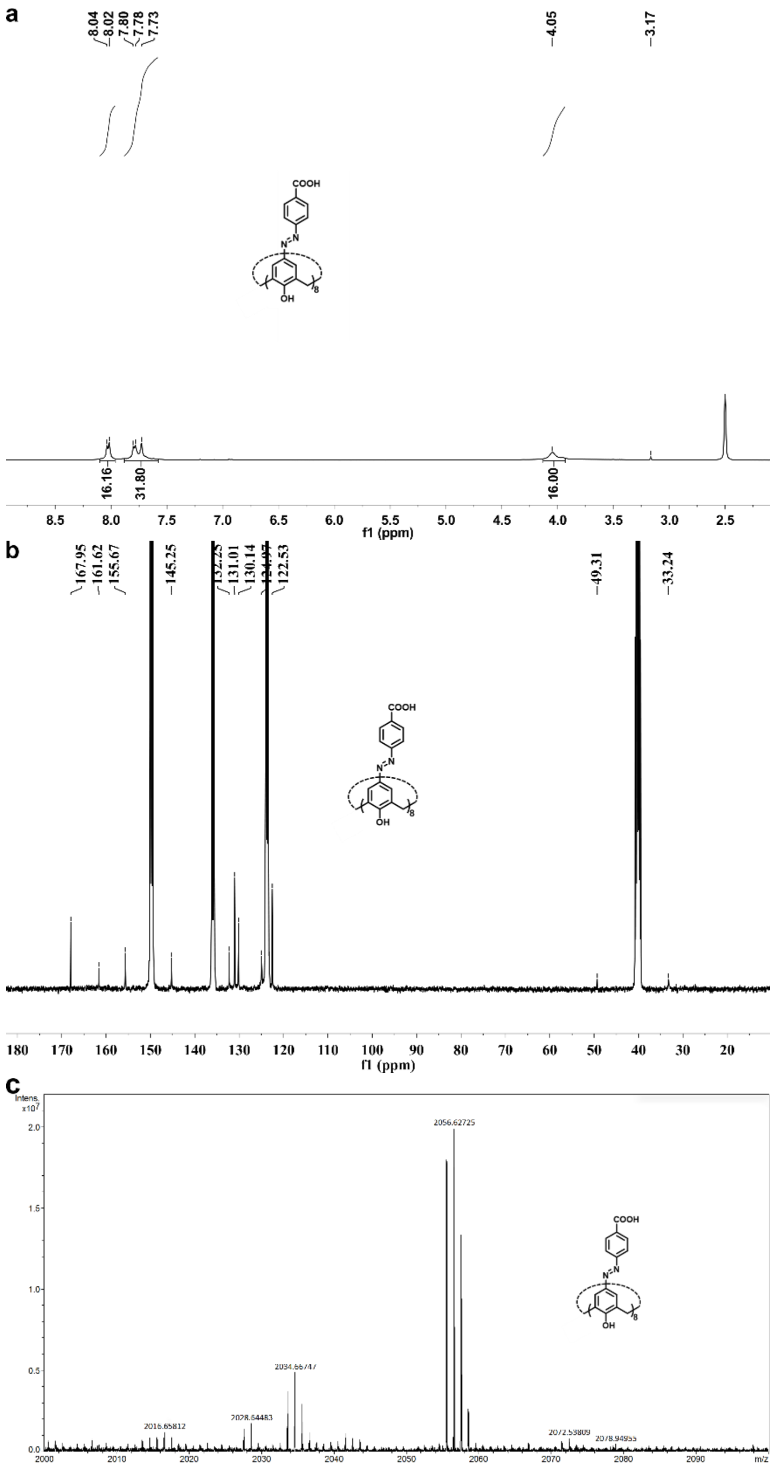

Figure S5. a) ^{1}H NMR spectrum of CAC8A in DMSO-d_6, 400 MHz, 25 °C. A signal (3.17 ppm) for methanol was observed, probably because methanol was encapsulated into the cavity of CAC8A. b) ^{13}C NMR spectrum of CAC8A in Pyridine-d_5 and DMSO-d_6, 1:1, v:v, 100 MHz, 25 °C. A signal (49.31 ppm) for methanol was observed. c) Mass spectrum (MALDI-TOF) of CAC8A.

Synthesis of NH_2C4A

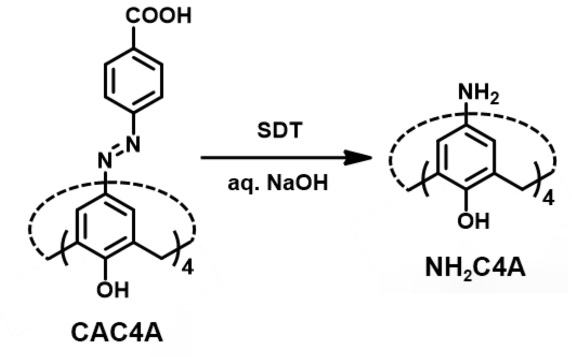

Scheme S2. The synthetic route of NH_2C4A

5,11,17,23-tetraamino-25,26,27,28-tetrahydroxycalix[4]arene (NH_2C4A).[10] CAC4A (1.76 g, 1.73 mmol) was dissolved in an aqueous NaOH solution (200 mL, 1%) and reduced with sodium hydrosulfite (7.0 g, 40 mmol) for 1 h at 90 °C to give a white suspension. The suspension was cooled rapidly to 20 °C, filtered, and washed with water, concentrated HCl and methanol. The formed precipitate was dried at room temperature under reduced pressure to obtain NH_2C4A in a 48% yield. ^1H NMR (400 MHz, D_2O) δ 7.14 (s, 8H, calix-Ar-H), 3.97 (s, 8H, Ar-CH_2-Ar) ppm.

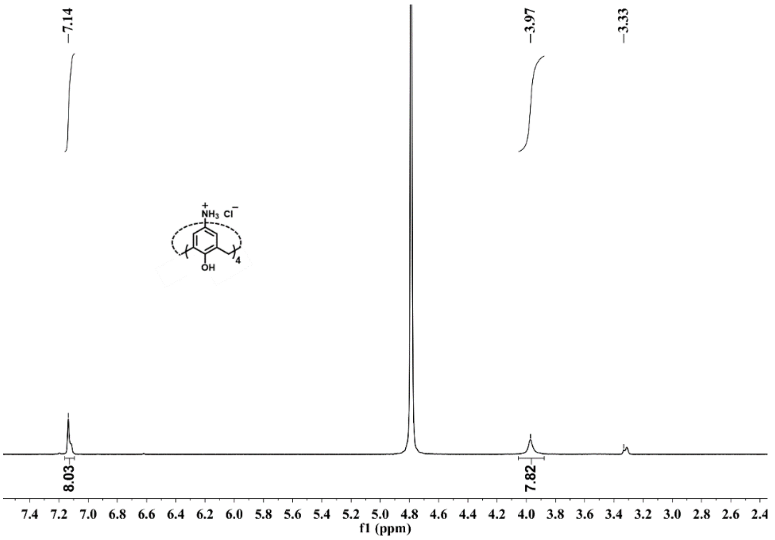

Figure S6. ^1H NMR spectrum of tetrahydrochloride of NH_2C4A in D_2O, 400 MHz, 25 °C. A signal (3.33 ppm) for methanol was observed, probably because methanol was encapsulated into the cavity of NH_2C4A.[1]

2.2 Fluorescence titrations

Direct fluorescence titrations of AQ4N with CACnAs

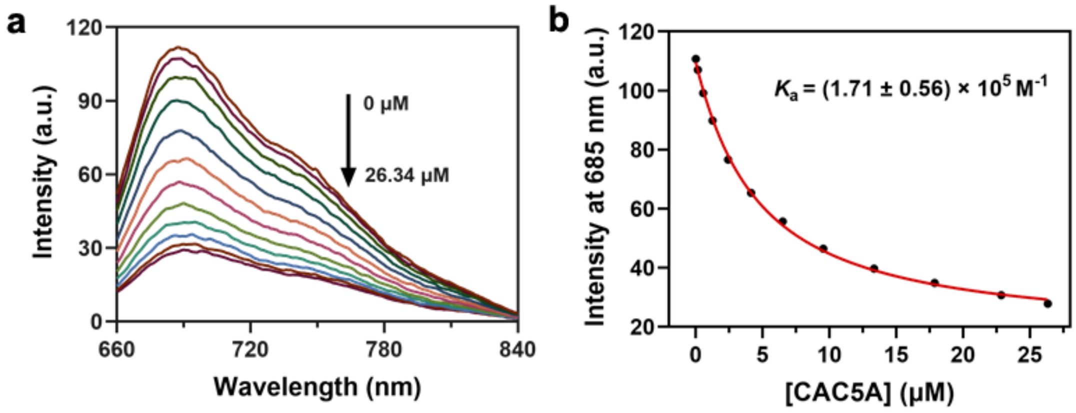

Figure S7. a) Direct fluorescence titration of AQ4N (2.00 μM) with CAC5A (up to 26.34 μM) in PBS buffer (10 mM, pH = 7.4) at 25 °C, λ_{ex} = 610 nm. b) The associated titration curve at λ_{em} = 685 nm was fitted according to the 1:1 binding stoichiometry.

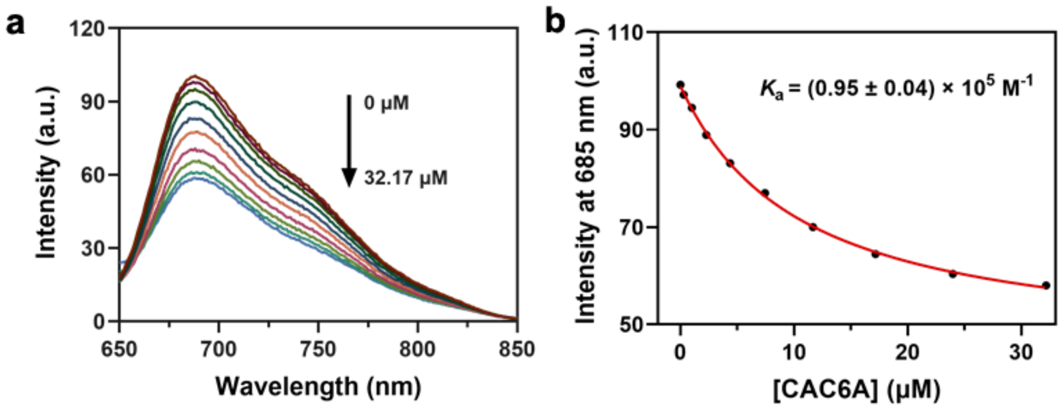

Figure S8. a) Direct fluorescence titration of AQ4N (2.00 μM) with CAC6A (up to 32.17 μM) in PBS buffer (10 mM, pH = 7.4) at 25 °C, λ_{ex} = 610 nm. b) The associated titration curve at λ_{em} = 685 nm was fitted according to the 1:1 binding stoichiometry.

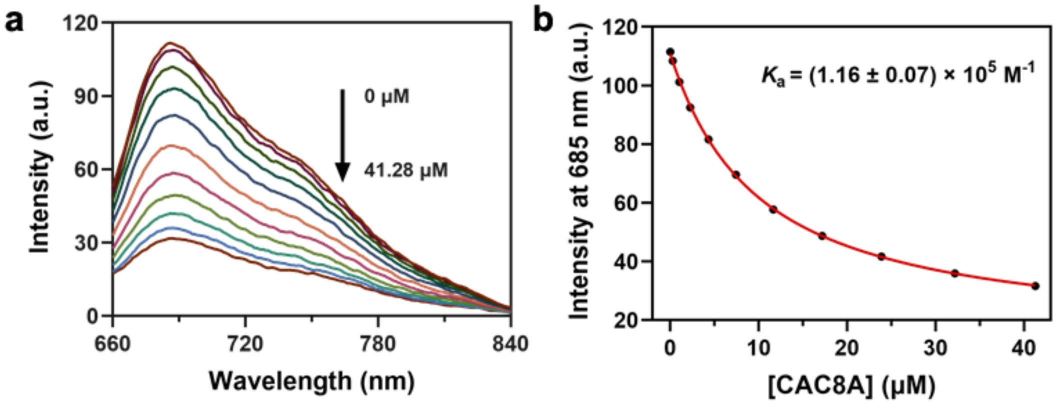

Figure S9. a) Direct fluorescence titration of AQ4N (2.00 μM) with CAC8A (up to 41.28 μM) in PBS buffer (10 mM, pH = 7.4) at 25 °C, λ_{ex} = 610 nm. b) The associated titration curve at λ_{em} = 685 nm was fitted according to the 1:1 binding stoichiometry.

Direct fluorescence titrations of AQ4 with CAC4A

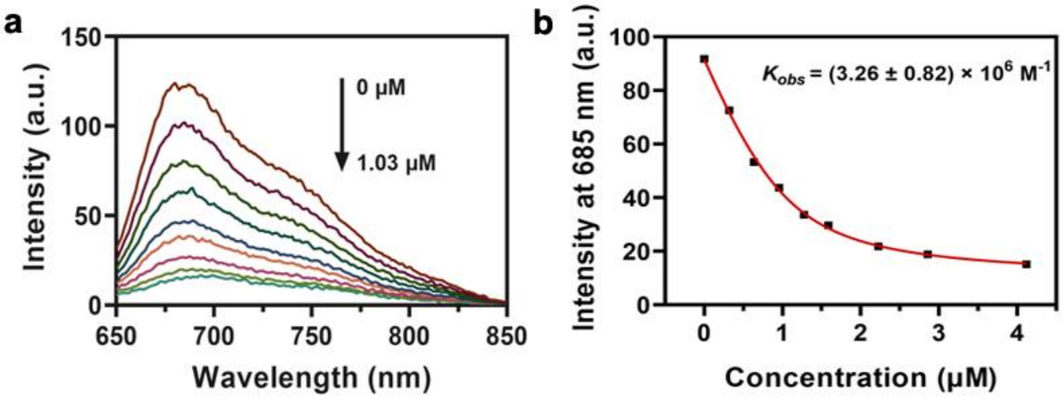

Figure S10. a) Direct fluorescence titration of AQ4 (1.0 μM) with CAC4A in PBS buffer (10 mM, pH = 7.4) at 25℃, λ_{ex} = 610 nm. b) The associated titration curve at λ_{em} = 685 nm was fitted according to the 1:1 binding stoichiometry using the concentration of calixarene binding site (calixarene concentration times 4) to obtain the K_{obs}.

The complexation mode between CAC4A and AQ4 is complicated because of the complexation-induced aggregation of AQ4.[11] One CAC4A can complex more than one AQ4, we speculate that one CAC4A can complex four AQ4 according to the inflection point at a quarter concentration of AQ4 of the direct fluorescence titration curve, that is one CAC4A has four binding sites towards AQ4. We assumed that these binding sites are identical and

independent, i.e., the affinity of any site does not depend on whether or not the other sites are occupied. We fitted the data according to the 1:1 binding stoichiometry using the concentration of calixarene binding site (calixarene concentration times 4) to obtain the obvious binding constant (K_{obs}), which is defined as a measure of the binding strength between a single AQ4 and a single binding site of CAC4A. Such an isodesmic simplification (equal-K model) is reasonably accepted in this system to obtain the K_{obs}.[12]

Direct fluorescence titration of AQ4N with NH_2C4A

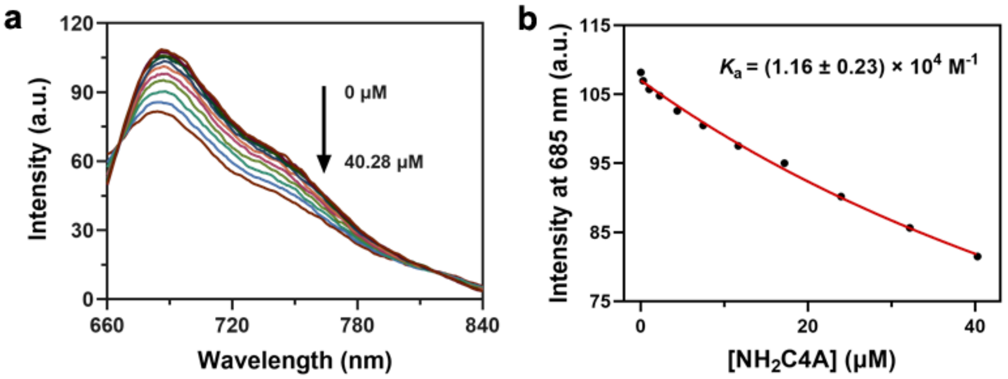

Figure S11. a) Direct fluorescence titration of AQ4N (2.00 μM) with NH_2C4A in PBS buffer (10 mM, pH = 7.4) at 25 °C, λ_{ex} = 610 nm. b) The associated titration curve at λ_{em} = 685 nm was fitted according to the 1:1 binding stoichiometry.

Direct fluorescence titration of AQ4 with NH_2C4A

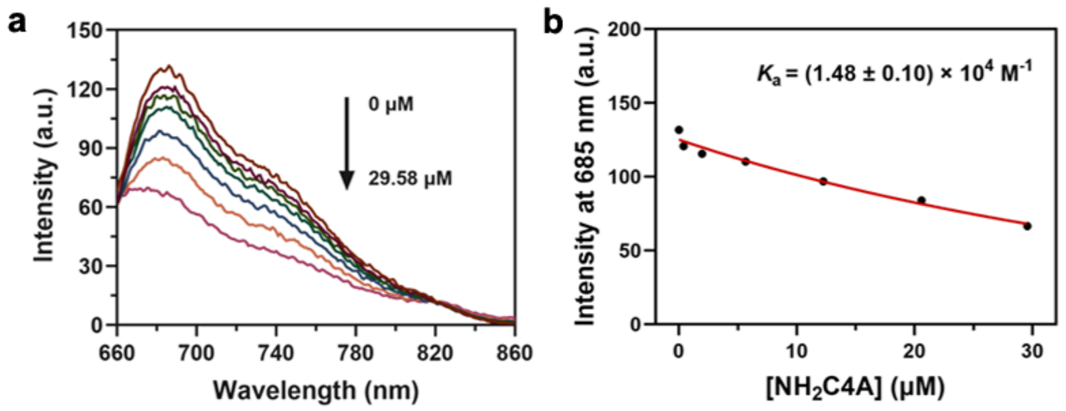

Figure S12. a) Direct fluorescence titration of AQ4 (1.0 μM) with NH_2C4A in PBS buffer (10 mM, pH = 7.4) at 25℃, λ_{ex} = 610 nm. b) The associated titration curve at λ_{em} = 685 nm was fitted according to the 1:1 binding stoichiometry.

2.3 UV-Vis spectra of CAC4A, AQ4N and CAC4A•AQ4N

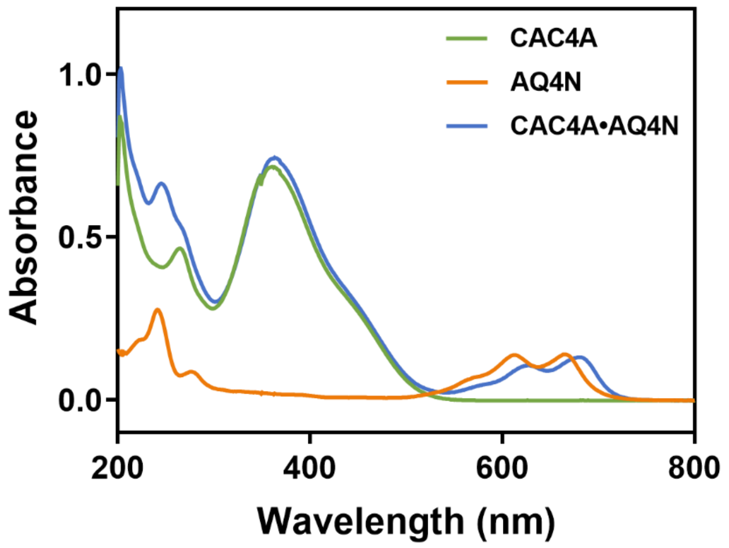

Figure S13. UV-Vis absorption spectra of AQ4N (10 μM), CAC4A (10 μM), and CAC4A•AQ4N (10/10 μM) in PBS buffer (10 mM, pH = 7.4).

2.4 Job's plot for the complexation of CAC4A•AQ4N

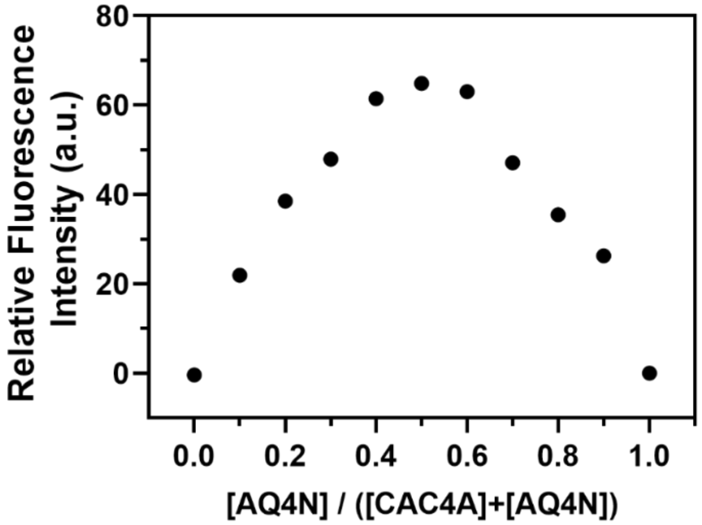

Figure S14. Job's plot for the complexation of AQ4N and CAC4A in PBS buffer (10 mM, pH = 7.4) at 25 °C, [AQ4N] + [CAC4A] = 2.00 μM.

2.5 The mechanism behind the strong binding between CAC4A and AQ4N

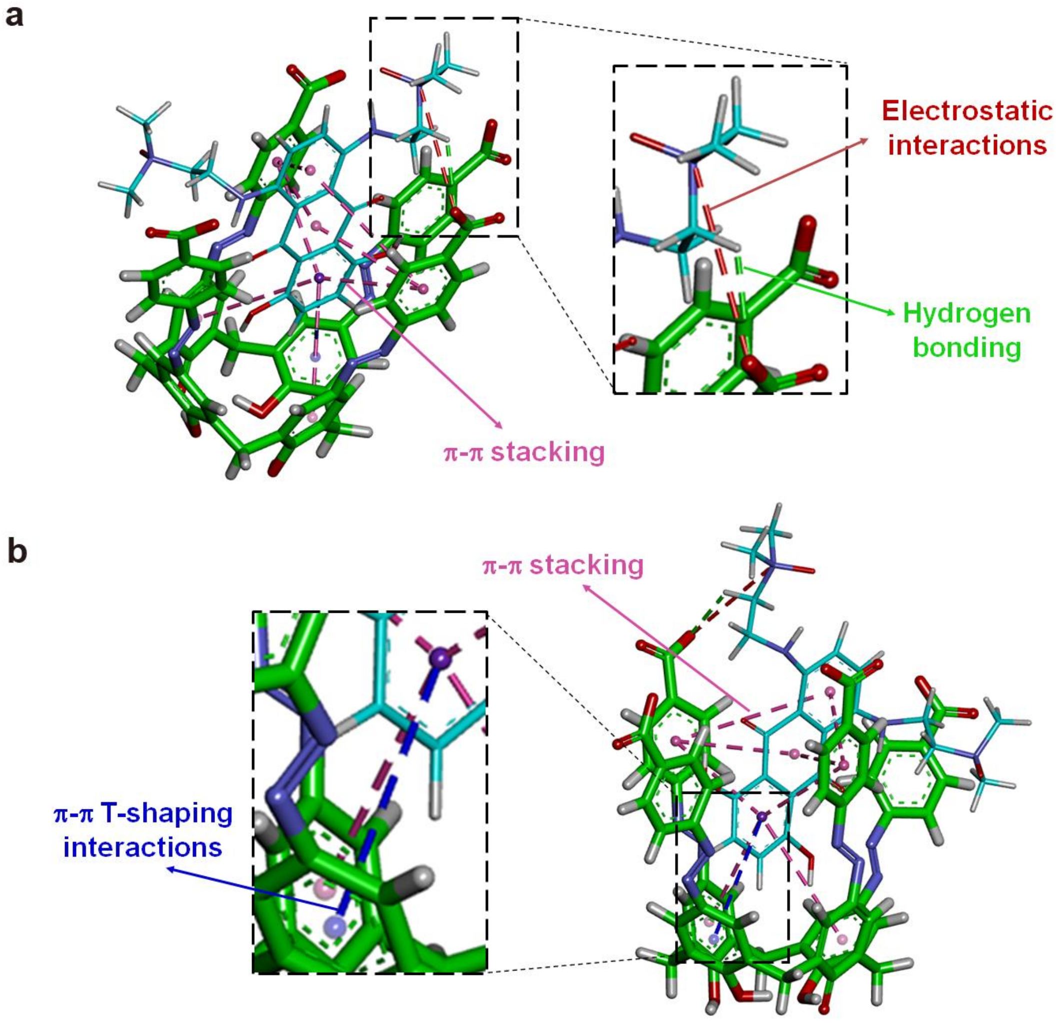

Figure S15. Interactions in the final pose of CAC4A•AQ4N complex after a 100 ns molecular dynamics simulation in different orientation. C atoms of CAC4A and AQ4N are shown in green and cyan, respectively. Oxygen and nitrogen atoms in this complex are colored by red and blue, respectively. π-π stacking and π-π T-shaping interactions are highlighted in pink and blue dotted lines, respectively. Electrostatic and hydrogen bonding interactions are colored by red and green dotted lines, respectively. The hydrogen-bonding criteria are (i) the angle C−H···O > 135° and (ii) the distance C···O < 3.5 Å.

2.6 The integrity of CAC4A, AQ4N and CAC4A•AQ4N in heme

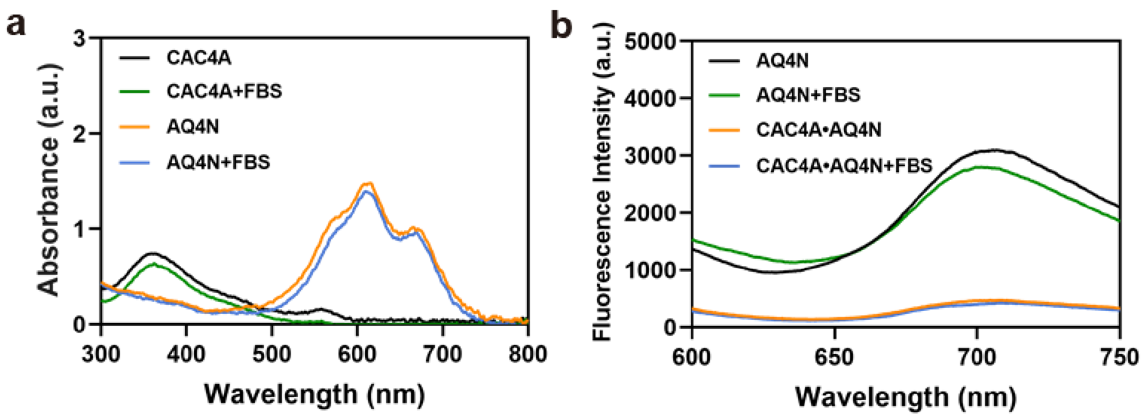

Figure S16. a) UV−vis absorption spectra of CAC4A and AQ4N with or without incubation of serum. b) Fluorescence spectrum of AQ4N and CAC4A•AQ4N with or without incubation of serum.

2.7 UV-Vis spectrum of AQ4N@DPPC

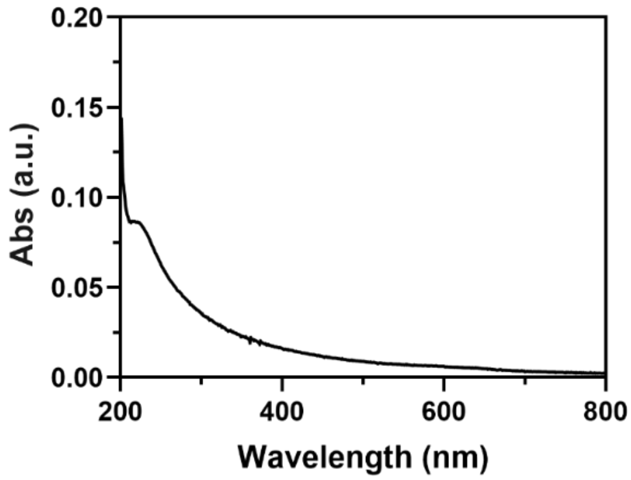

Figure S17. UV-Vis spectrum of AQ4N@DPPC in PBS buffer (10 mM, pH = 7.4) at 25 °C.

2.8 Hypoxic response of CAC4A

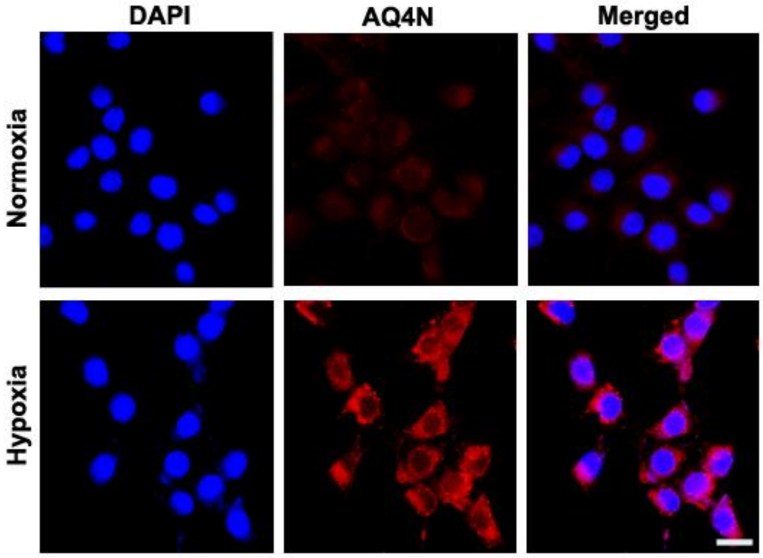

Figure S18. Fluorescence images of 4T1 cells after incubation with CAC4A•AQ4N (10/10 μM) for 6 h under normoxia and hypoxia conditions. Cell nucleus were stained with DAPI. Scale bar, 50 μm.

2.9 Cellular uptake

The time-dependent cellular internalization of CAC4A•AQ4N

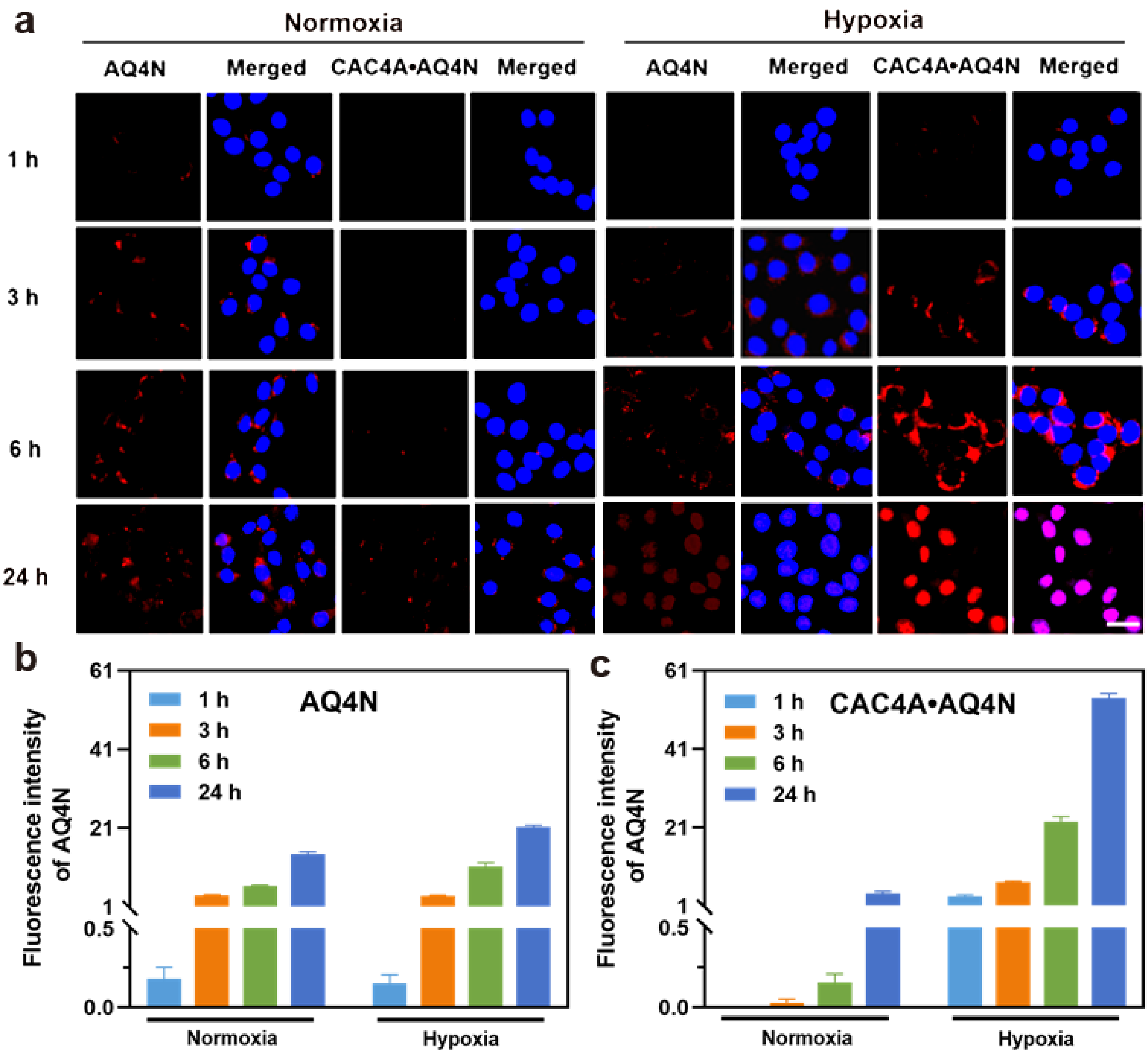

Figure S19. a) Fluorescence images showing the cellular uptake of AQ4N and CAC4A•AQ4N by 4T1 cells at 1, 3, 6 and 24 h under normoxia environment and hypoxia environment. scale bar, 50 μm. Quantitative analysis of fluorescence intensity of AQ4N in 4T1 cells treated with b) AQ4N and c) CAC4A•AQ4N at different times. The data was shown as mean ± s.d. ($n = 20$).

The endocytosis mechanisms of CAC4A•AQ4N

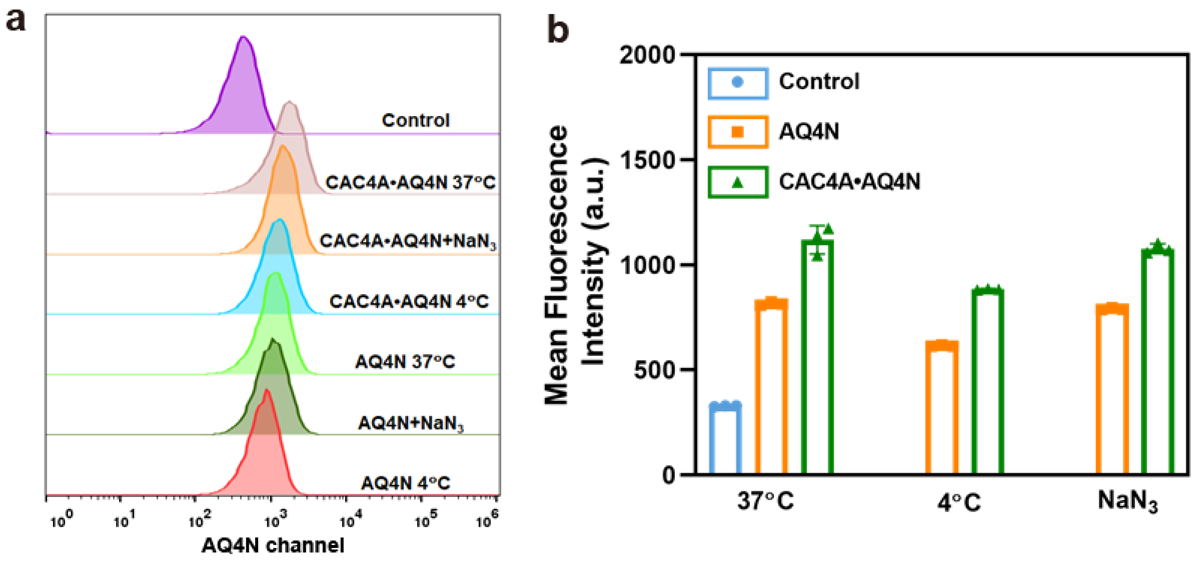

Figure S20. a) Investigation of the cellular uptake mechanism of AQ4N and CAC4A•AQ4N using the treatment of inhibit endocytosis (low temperature (4°C) and NaN_3 (10 mM)). b) Quantitative analysis of fluorescence changes in cells after treated with corresponding inhibitors. The data was shown as mean ± s.d. (n = 3).

2.10 The cytotoxicity of CAC4A•AQ4N

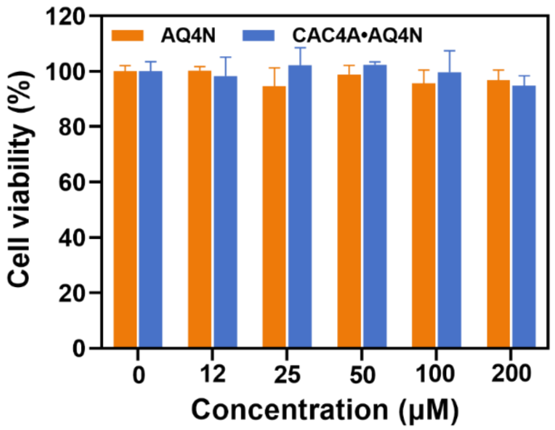

Figure S21. Cell viabilities of 4T1 cells treated with different concentrations of AQ4N and CAC4A•AQ4N. The data are shown as mean ± s.d. (n = 3).

2.11 *In vitro* sensitization efficiency study

Colony formation assay

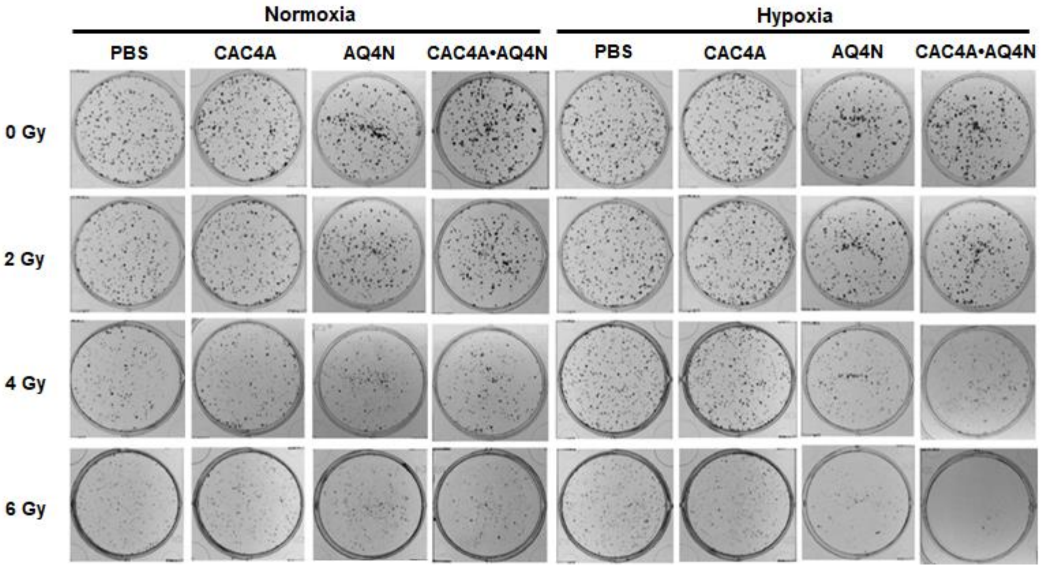

Figure S22. Colony formation of 4T1 cells treated with PBS, CAC4A, AQ4N, CAC4A•AQ4N at radiation dosage of 0, 2, 4 and 6 Gy.

Table S1. The SER value of different treatments.

Samples	PBS	CAC4A	AQ4N	CAC4A•AQ4N
SER value (Normoxia)	–	0.985	1.317	1.665
SER value (Hypoxia)	0.992	0.991	1.528	2.349

Immunofluorescent γ-H2AX staining

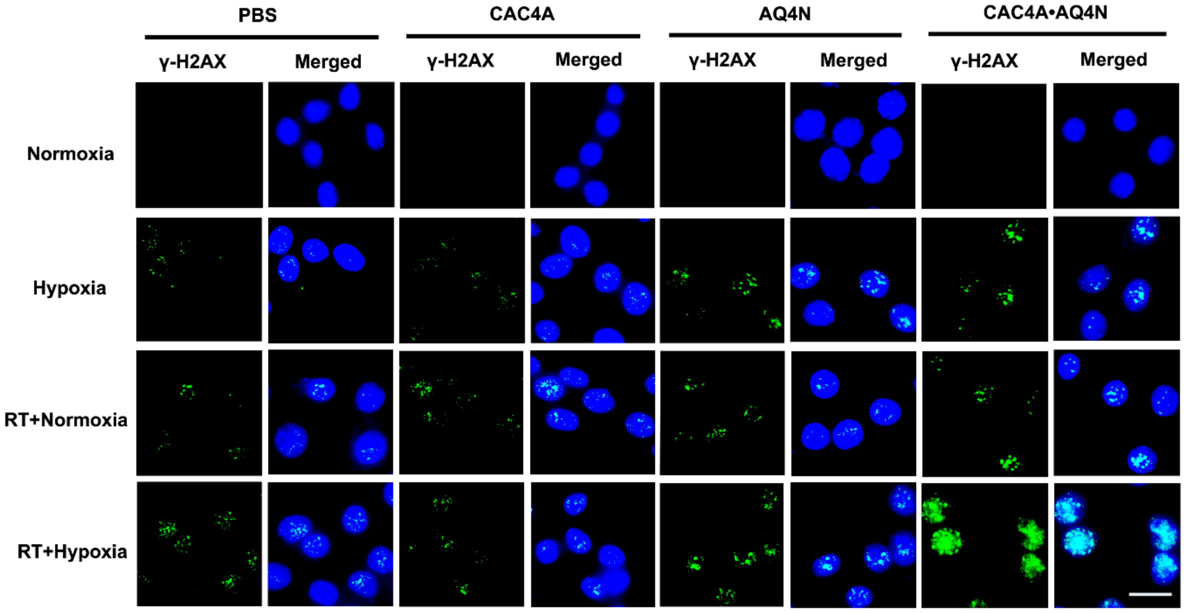

Figure S23. Immunofluorescent γ-H2AX staining of 4T1 cells treated with different interventions at radiation dosage of 6 Gy. Scale bar, 50 μm.

Flow cytometric analysis of cell apoptosis

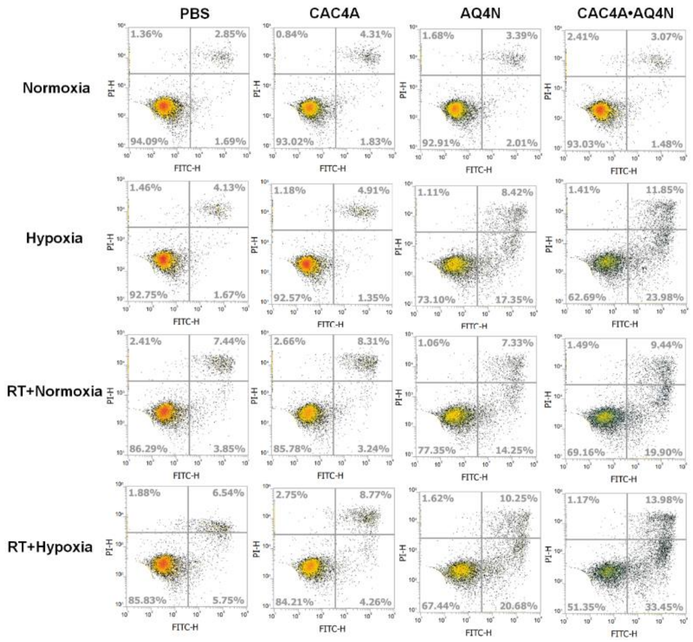

Figure S24. Flow cytometric analysis of apoptosis ratio of 4T1 cells treated with different interventions at radiation dosage of 6 Gy.

2.12 Biodistribution of CAC4A•AQ4N

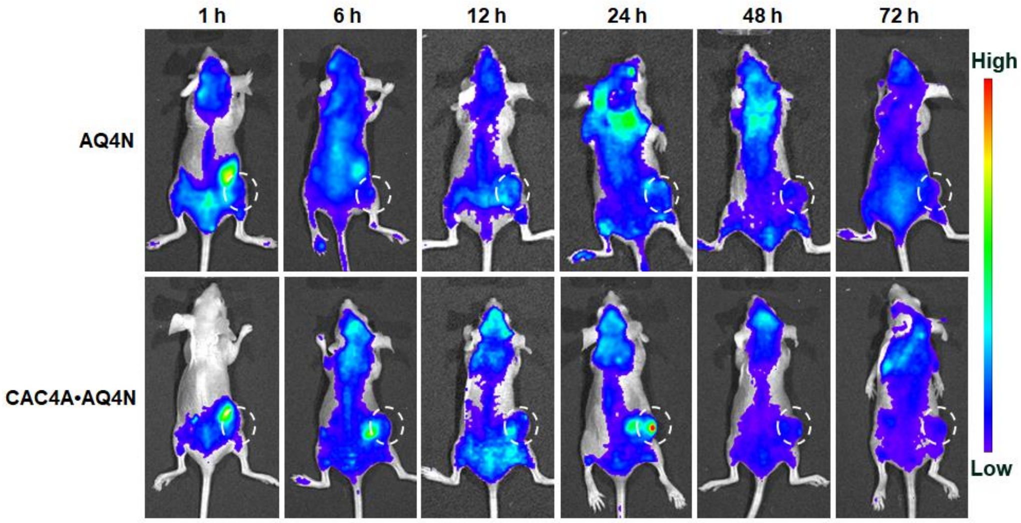

Figure S25. *In vivo* fluorescence images of 4T1-bearing mice after intravenous administration of AQ4N and CAC4A•AQ4N at 1, 6, 12, 24, 48 and 72 h post-injection. Tumors tissues were circled by white dash line.

2.13 TUNEL staining of tumor slices from mice treated with CAC4A•AQ4N

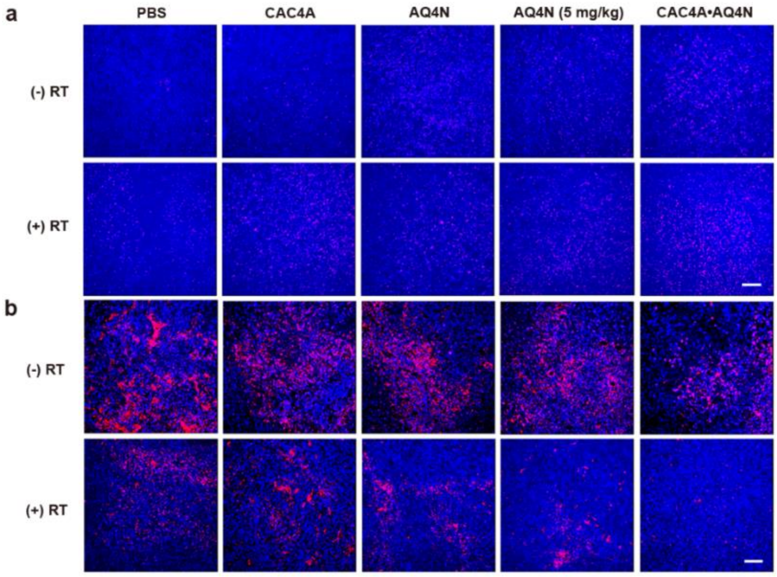

Figure S26. a) TUNEL staining of tumor slices from mice treated with PBS, CAC4A, AQ4N, AQ4N (5 mg/kg) and CAC4A•AQ4N with/without γ-ray irradiation (radiation dosage: 6 Gy). Scale bar, 100 μm. b) Immunofluorescence proliferating nucleus proteins Ki-67 staining

analysis of tumor sections from mice in different groups. Scale bar, 100 μm. Cell nucleus were stained with DAPI.

2.14 Safety evaluation of CAC4A•AQ4N

The biocompatibility of CAC4A•AQ4N on normal cells

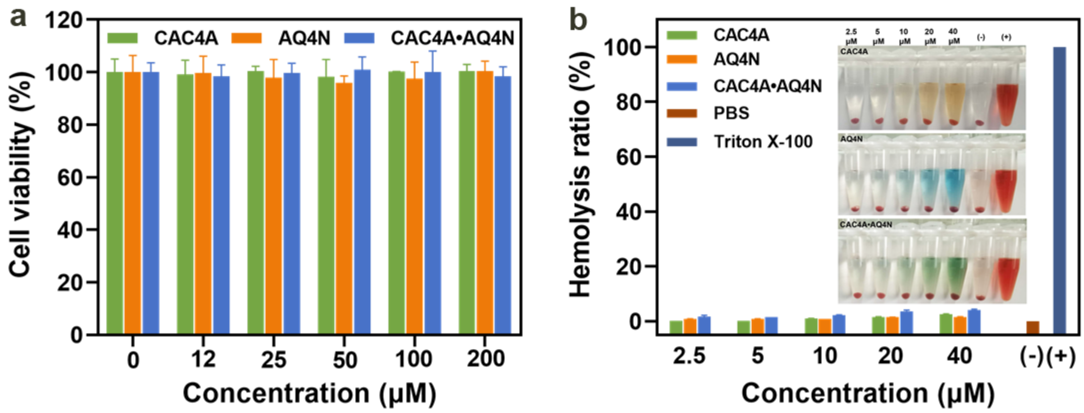

Figure S27. a) Cell viabilities of 3T3 cells treated with different concentrations of CAC4A, AQ4N and CAC4A•AQ4N. b) Hemolysis analysis of CAC4A, AQ4N and CAC4A•AQ4N at predetermined concentrations. Red blood cells incubated with PBS and 0.1% Triton X-100 (diluted with PBS) were used as negative (−) and positive (+) control, respectively. The data are shown as mean ± s.d. (n = 3).

Body weight changes of the mice treated with CAC4A•AQ4N

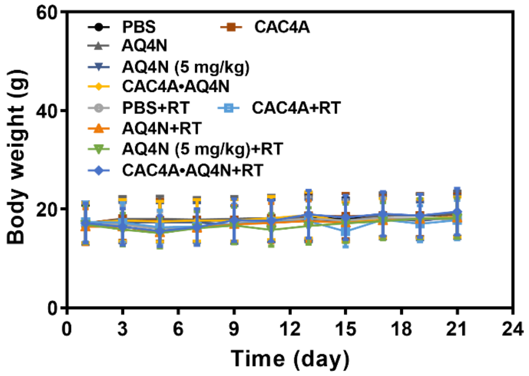

Figure S28. The changes in body weight of mice treated with PBS, CAC4A, AQ4N, AQ4N (5 mg/kg) and CAC4A•AQ4N with/without γ-ray irradiation (radiation dosage: 6 Gy). The data was shown as mean ± s.d. (n = 5).

H&E stains of the major organs from mice treated with CAC4A•AQ4N

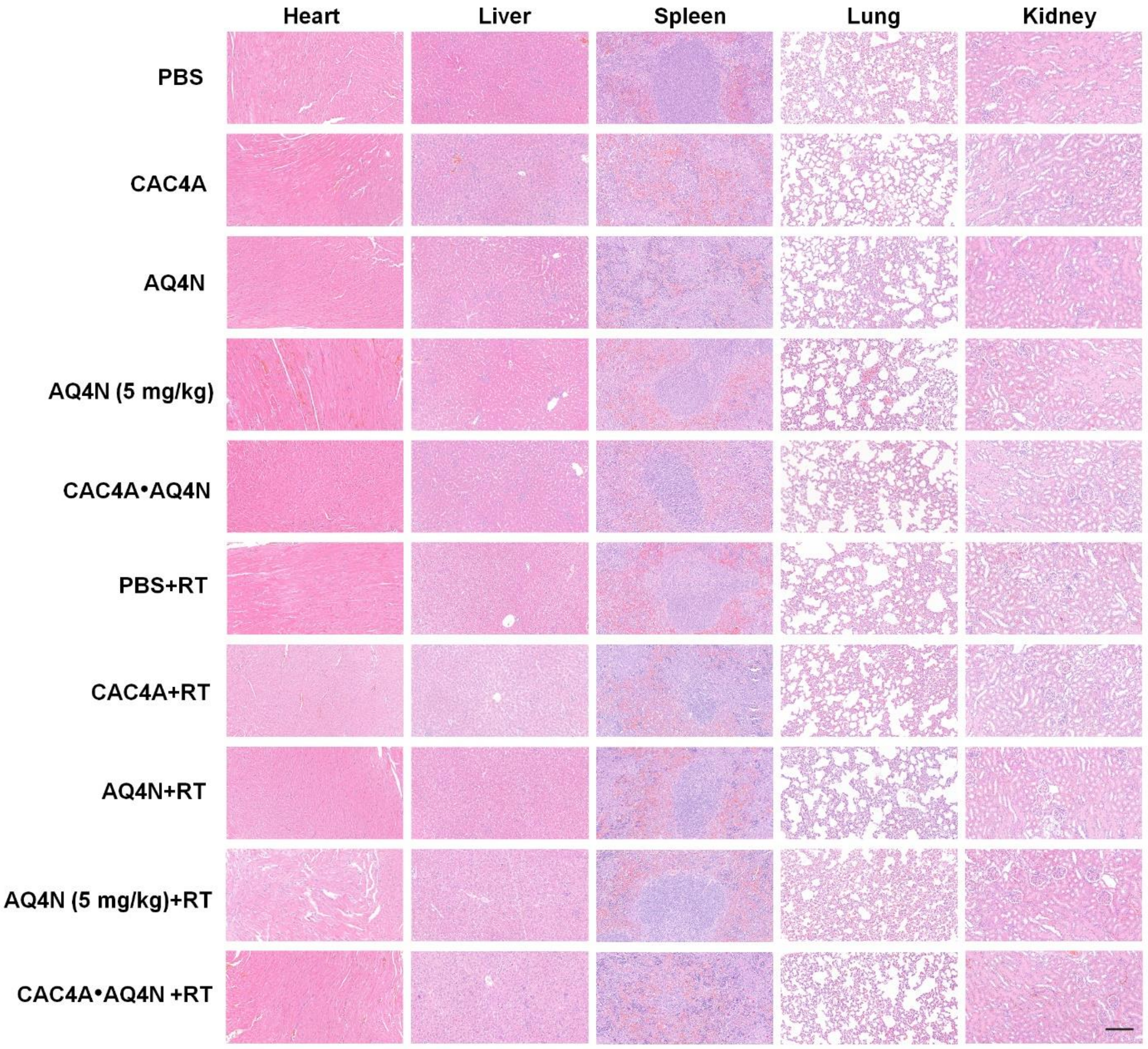

Figure S29. H&E staining image analysis of major organs (heart, liver, spleen, lung and kidney) from mice in different groups. Scale bar, 100 μm.

Blood biochemistry and hematology assay

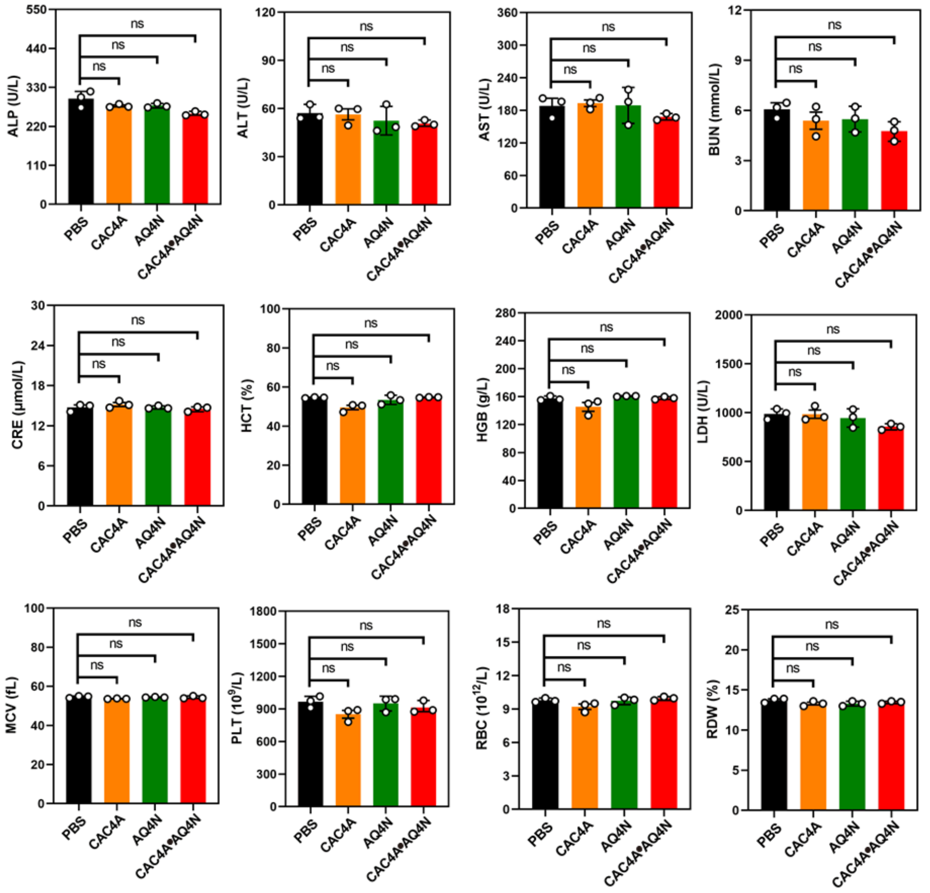

Figure S30. Blood biochemistry and hematology assay of mice treated with PBS, CAC4A, AQ4N and CAC4A•AQ4N. The data was shown as mean ± s.d. (n = 3). P values were calculated by Student's t test: ns represents "no significant difference".

2.15 Binding data of CAC4A with various drug molecules

Table S2. Binding affinities (K_a) of CAC4A with 9 drugs.

Guest	K_a [M^{-1}]	Guest	K_a [M^{-1}]	Guest	K_a [M^{-1}]
paclitaxel[a]	1.6×10^6	docetaxel[a]	4.6×10^5	doxorubicin[a]	1.6×10^8
pirarubicin[a]	5.0×10^7	camptothecin[a]	2.0×10^6	irinotecan[a]	5.4×10^6
hydroxycamptothecin[a]	1.1×10^5	topotecan[a]	1.7×10^5	tamoxifen[a]	2.0×10^5

[a] Data from Ref. (1)

Molecular structures of hydroxychloroquine and lovastatin

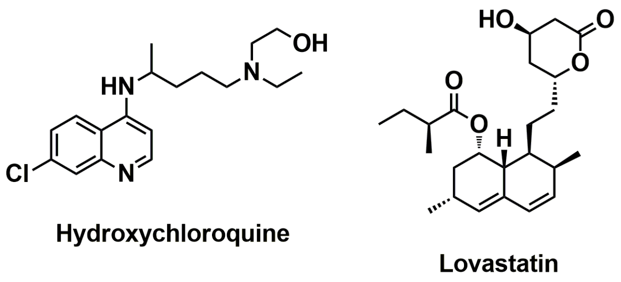

Competitive fluorescence titration of RhB•CAC4A with hydroxychloroquine

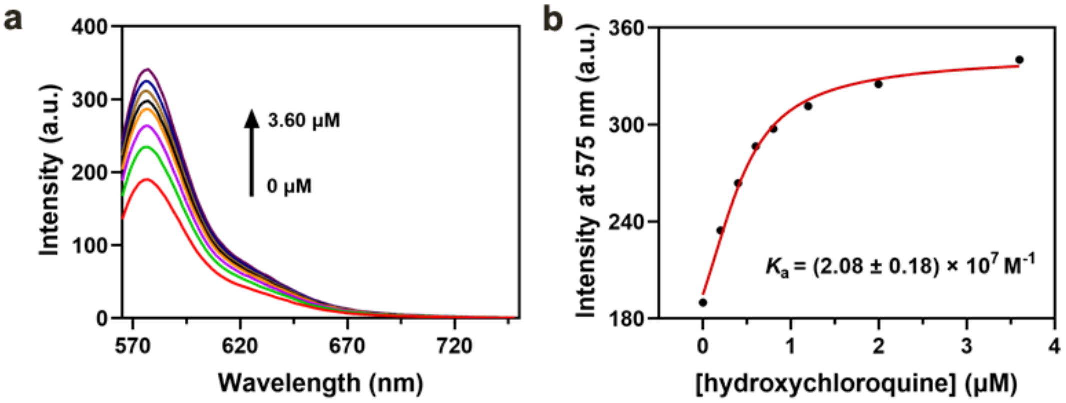

Figure S31. a) Competitive fluorescence titration of CAC4A•RhB (0.50/0.50 µM) with hydroxychloroquine (up to 3.60 µM) in PBS buffer (10 mM, pH = 7.4) at 25 °C, λ_{ex} = 554 nm. b) The associated titration curve at λ_{em} = 575 nm was fitted according to a 1:1 binding stoichiometry.

Competitive fluorescence titration of RhB•CAC4A with lovastatin

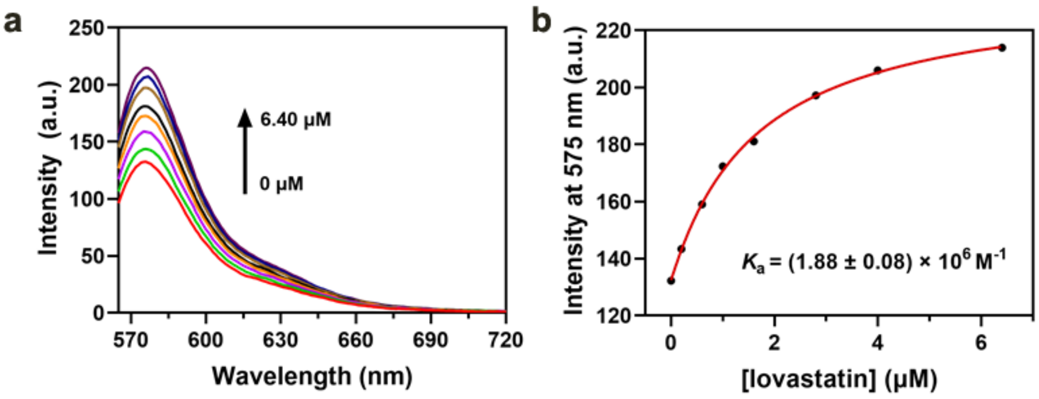

Figure S32. a) Competitive fluorescence titration of CAC4A•RhB (0.30/0.30 μM) with lovastatin (up to 6.40 μM) in PBS buffer (10 mM, pH = 7.4) at 25 °C, λ_{ex} = 554 nm. b) The associated titration curve at λ_{em} = 575 nm was fitted according to a 1:1 binding stoichiometry.

2.16 Hypoxia response of CAC4A•DOX and CAC4A•THP

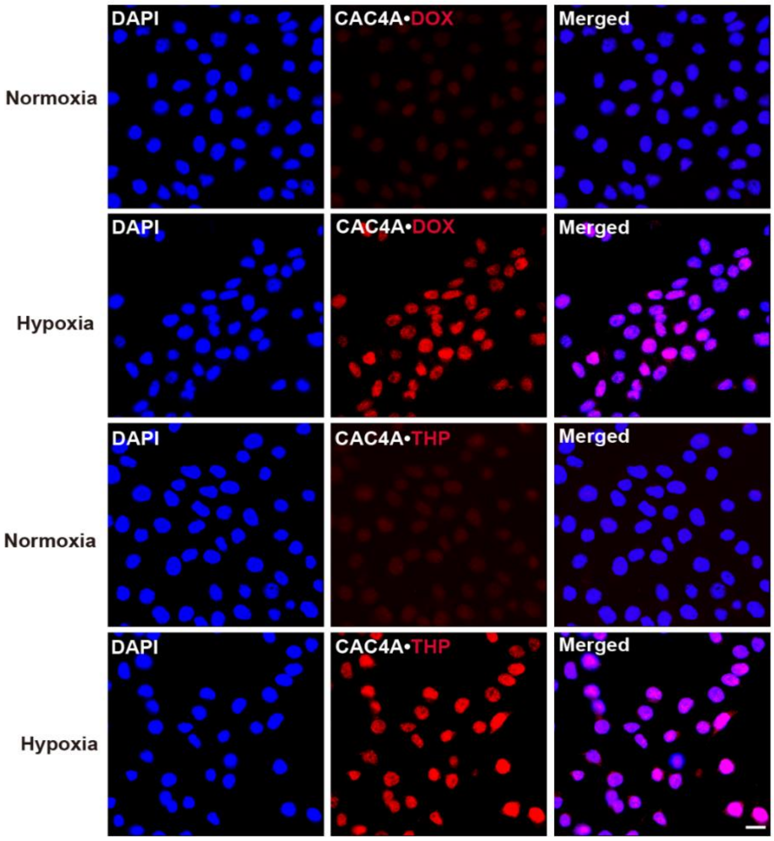

Figure S33. Fluorescence images of 4T1 cells after incubation with CAC4A•DOX and CAC4A•THP for 6 h under normoxia and hypoxia conditions. Cell nucleus were stained with DAPI. Scale bar, 50 μm.

2.17 Cellular uptake of CAC4A•DOX and CAC4A•THP

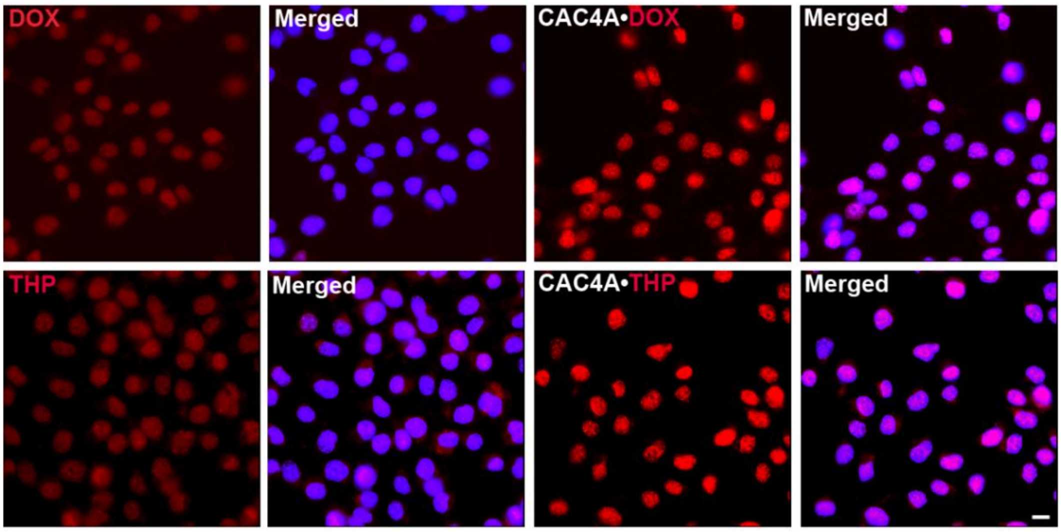

Figure S34. Fluorescence images showing the cellular uptake of free drugs (DOX and THP) and the complex (CAC4A•DOX and CAC4A•THP) by 4T1 cells at 6 h under hypoxia environment. scale bar, 50 μm.

2.18 The radiosensitizing efficacy of supramolecular complexes

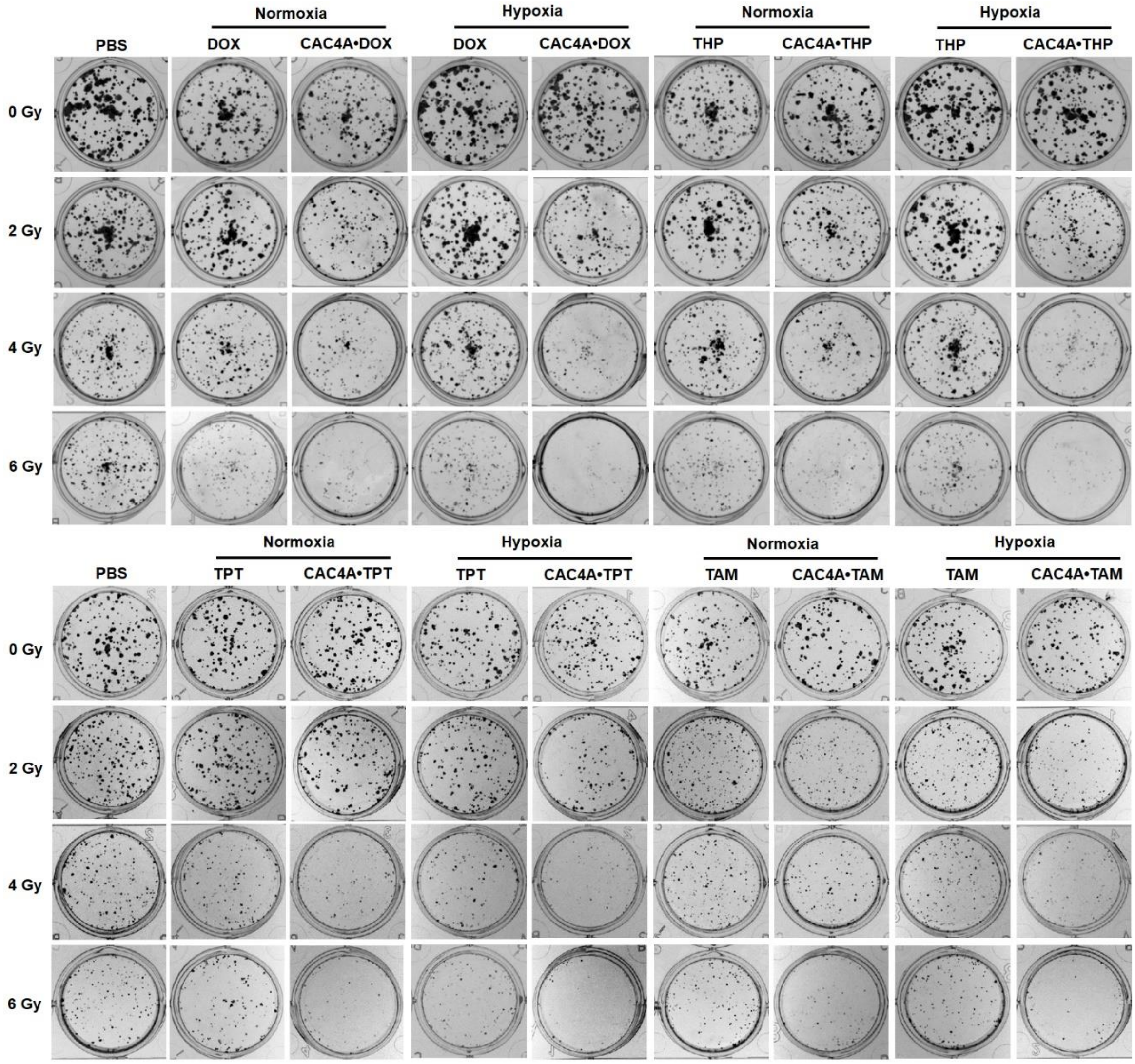

Figure S35. Colony formation of 4T1 cells treated with radiosensitive small molecule drugs (DOX, THP, TPT and TAM) and supramolecular complexes (CAC4A•DOX, CAC4A•THP, CAC4A•TPT and CAC4A•TAM) at radiation dosage of 0, 2, 4 and 6 Gy.

Table S3. The SER value of different treatments.

Samples	SER value (Normoxia)	SER value (Hypoxia)
DOX	1.77	1.62
CAC4A•DOX	2.21	2.62
THP	1.53	1.33
CAC4A•THP	1.85	2.31
TPT	1.34	1.29
CAC4A•TPT	1.58	1.92
TAM	1.33	1.20
CAC4A•TAM	1.46	1.89

3. References

[1] T.-X. Zhang, Z.-Z. Zhang, Y.-X. Yue, X.-Y. Hu, F. Huang, L. Shi, Y. Liu, D.-S. Guo, *Adv. Mater.* **2020**, *32*, 1908435.

[2] E. Hatcher, O. Guvench, M. A. Jr, *J. Chem. Theory Comput.* **2009**, *5*, 1315.

[3] a) K. Vanommeslaeghe, E. Hatcher, C. Acharya, S. Kundu, S. Zhong, J. Shim, E. Darian, O. Guvench, P. Lopes, I. Vorobyov, J. A. D. Mackerell, *J. Comput. Chem.* **2010**, *31*, 671; b) O. Guvench, S. N. Greene, G. Kamath, J. W. Brady, R. M. Venable, R. W. Pastor, A. D. M. Jr, *J. Comput. Chem.* **2008**, *29*, 2543; c) O. Guvench, E. R. Hatcher, R. M. Venable, R. W. Pastor, M. K. Jr, *J. Chem. Theory Comput.* **2009**, *5*, 2353.

[4] W. L. Jorgensen, J. Chandrasekhar, J. D. Madura, R. W. Impey, M. L. Klein, *J. Comput. Phys.* **1983**, *79*, 926.

[5] W. Humphrey, A. Dalke, K. Schulten, *J. Mol. Graph. Model.* **1996**, *14*, 33.

[6] L. Feng, L. Cheng, Z. Dong, D. Tao, T. E. Barnhart, W. Cai, M. Chen, Z. Liu, *ACS nano* **2017**, *11*, 927.

[7] a) P. I. Creeke, F. Dibari, E. Cheung, T. V. D. Briel, E. Kyroussis, A. J. Seal, *J. Nutr.* **2007**, *137*, 2013; b) N. Psychogios, D. D. Hua, J. Peng, A. C. Guo, R. Mandal, S. Bouatra,

I. Sinelnikov, R. Krishnamurthy, R. Eisner, B. Gautam, N. Young, J. Xia, C. Knox, E. Dong, P. Huang, Z. Hollander, T. L. Pedersen, S. R. Smith, F. Bamforth, R. Greiner, B. McManus, J. W. Newman, T. Goodfriend, D. S. Wishart, *PLoS ONE* **2011**, *6*, e16957; c) R. A. Harkness, S. B. Coade, A. D. B. Webster, *Clin. Chim. Acta* **1984**, *143*, 91; d) H. Ono, A. Sakamoto, N. Sakura, *Clin. Chim. Acta* **2001**, *312*, 227; e) D. J. Llewellyn, K. M. Langa, R. P. Friedland, I. A. Lang, *Curr. Alzheimer Res.* **2010**, *7*, 91.

[8] Y. Yong, X. Cheng, T. Bao, M. Zu, L. Yan, W. Yin, C. Ge, D. Wang, Z. Gu, Y. Zhao, *ACS Nano* **2015**, *9*, 12451.

[9] Y. Morita, T. Agawa, E. Nomura, H. Taniguchi, *J. Org. Chem.* **1992**, *57*, 3658.

[10] V. Rudzevich, O. Kasyan, A. Drapailo, M. Bolte, D. Schollmeyer, V. Bohmer, *Chem.-Asian J.* **2010**, *5*, 1347.

[11] D. S. Guo, B. P. Jiang, X. Wang, Y. Liu, *Org. Biomol. Chem.* **2012**, *10*, 720.

[12] G. Ghale, A. G. Lanctot, H. T. Kreissl, M. H. Jacob, H. Weingart, M. Winterhalter, W. M. Nau, *Angew. Chem.* **2014**, *53*, 2762.
